# Supplementary material for: Evolution of iGluR ligand specificity, polyamine regulation, and ion selectivity inferred from a placozoan epsilon receptor
Source: Commun Biol. 2025 Jul 3;8:994. doi: 10.1038/s42003-025-08402-3 (PMC12226733; doi:10.1038/s42003-025-08402-3)
Supplement: Supplementary file 2 — Supplementary Information [file 42003_2025_8402_MOESM2_ESM.pdf]

Evolution of iGluR ligand specificity, polyamine regulation, and ion selectivity inferred from a placozoan epsilon receptor

Anhadvir Singh[1], Boris S. Zhorov[2,3,†], Luis A. Yanez-Guerra[4,5,†], Alessandra Aleotti[6,†], Chloe C. Koens[7,†], C. Defne Yanartas[1], Yunqi Song[1], Federico Javier Miguez Cabello[8], Derek Bowie[8], and Adriano Senatore[1,*]

[1]Department of Biology, University of Toronto Mississauga, Mississauga Ontario, L5L 1C6, Canada.

[2]Department of Biochemistry and Biomedical Sciences, McMaster University, Hamilton Ontario, L8S 4K1, Canada.

[3]Sechenov Institute of Evolutionary Physiology & Biochemistry, Russian Academy of Sciences, Saint Petersburg 194223, Russian Federation.

[4]School of Biology, University of Southampton, Southampton, UK.

[5]Institute for Life Sciences, University of Southampton, Southampton, UK.

[6]Clinical and Experimental Sciences, Faculty of Medicine, University of Southampton, Southampton, UK.

[7]Integrated Program in Neuroscience, McGill University, Montreal Quebec, H3G 0B1, Canada.

[8]Department of Pharmacology & Therapeutics, McGill University, Montreal Quebec, H3G 0B1, Canada.

***Corresponding author: Adriano Senatore**

E-mail: adriano.senatore@utoronto.ca

Tel. (905)569-4322

[†]These authors contributed equally

Supplementary figures 1 to 9 – pages 2 to 10
Supplementary Tables 1 to 5 – pages 11 to 15
References – page 16

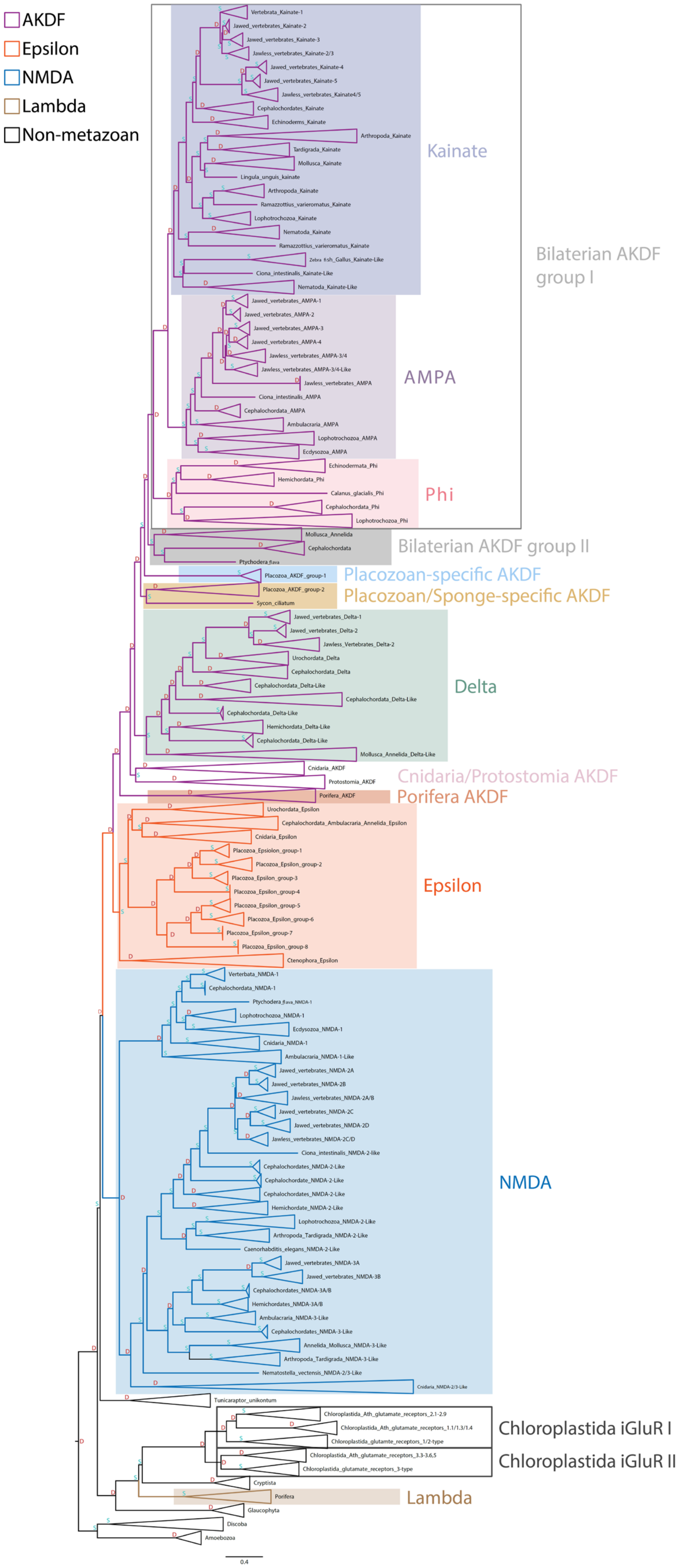

Supplementary Figure 1. Expanded GeneRax tree of iGluR protein sequences. The letters D (red) and S (cyan) denote predicted duplication and speciation and events, respectively, predicted by the GeneRax software [1].

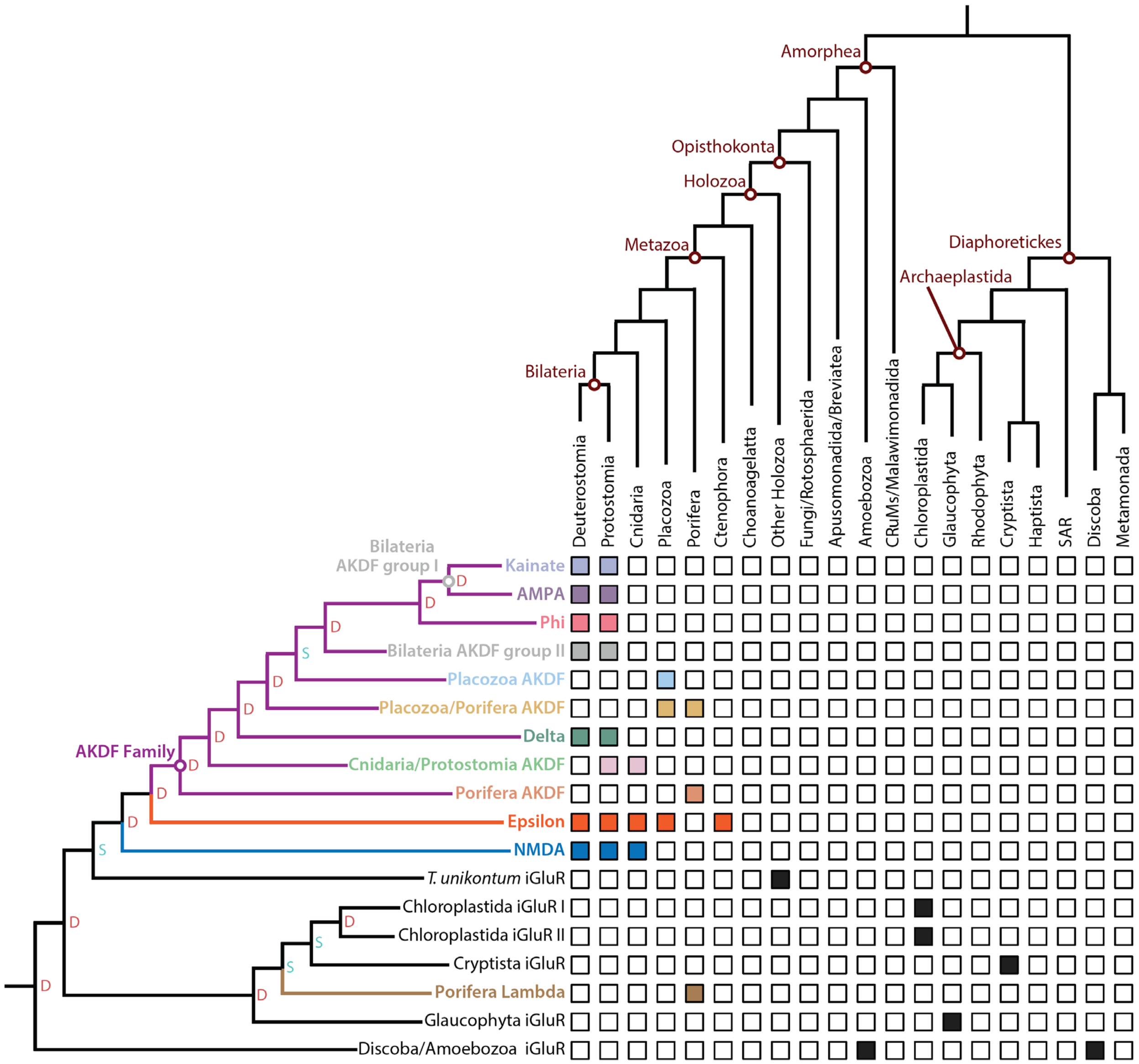

Supplementary Figure 2. Illustration denoting presence/absence of phylogenetically distinct iGluR types in metazoans and select non-metazoan eukaryotes. The letters D (red) and S (cyan) denote predicted duplication and speciation and events, respectively, predicted by the GeneRax software [1].

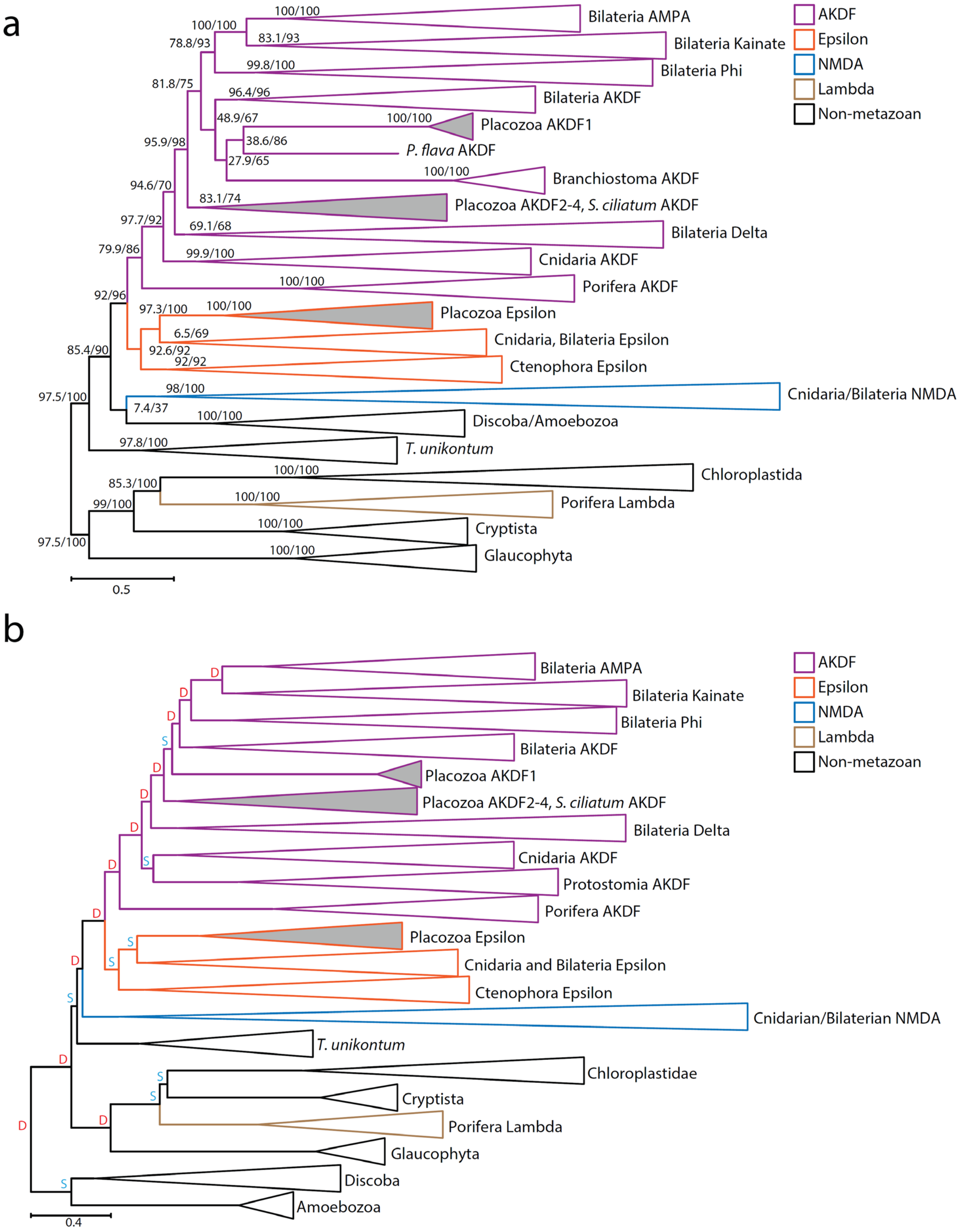

Supplementary Figure 3. Comparison of the GeneRax species aware tree with maximum likelihood iGluR protein phylogeny. a Maximum likelihood iGluR tree used as starting tree for gene tree to species tree reconciliation. The tree was inferred using IQ-TREE2 [2] with the evolutionary model Q.pfam+I+R10. Branch supports are percentages for 1000 replicates for the Shimodaira–Hasegawa-like approximate likelihood ratio test (SH-aLRT) and 1000 replicates for Ultrafast Bootstrap (UFB), respectively. **b** Copy of species aware phylogenetic tree shown in Figure 1a. The letters D (red) and S (cyan) denote predicted duplication and speciation and events, respectively, predicted by the GeneRax software [1].

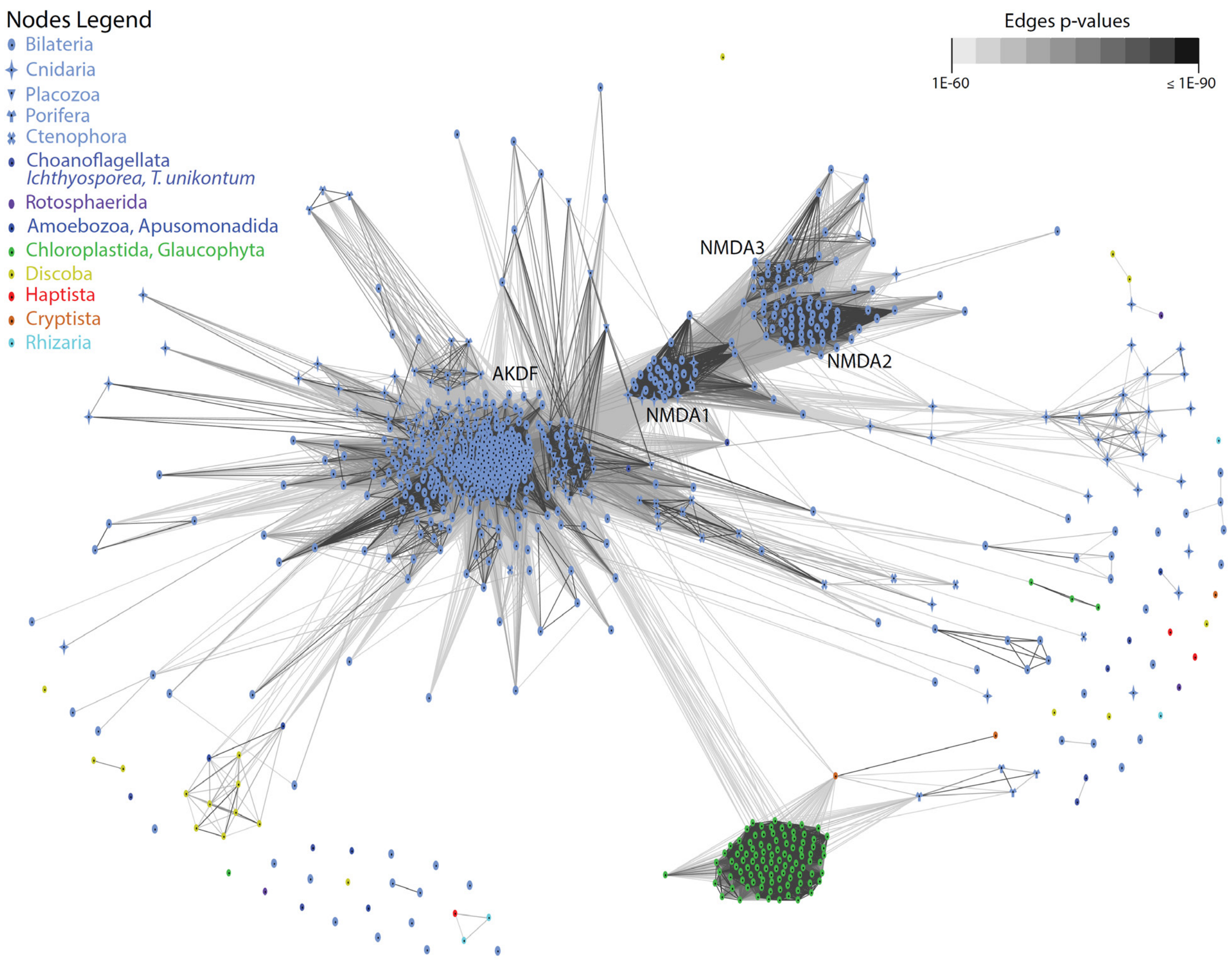

Supplementary Figure 4. Clustering of iGluR sequences with CLANS. Dots represent sequences, colour-coded by species group. Connecting lines represent BLAST scores. Clustering is shown at p-value 1.00E-60, which was the threshold utilized to delineate the iGluR family. A large cluster of animal iGluRs (light blue) is connected to a Chloroplastida iGluR cluster (green). Four sponge sequences connect to the latter but not to the former. A cluster of Amoebozoa/Discoba sequences connect to the animal cluster.

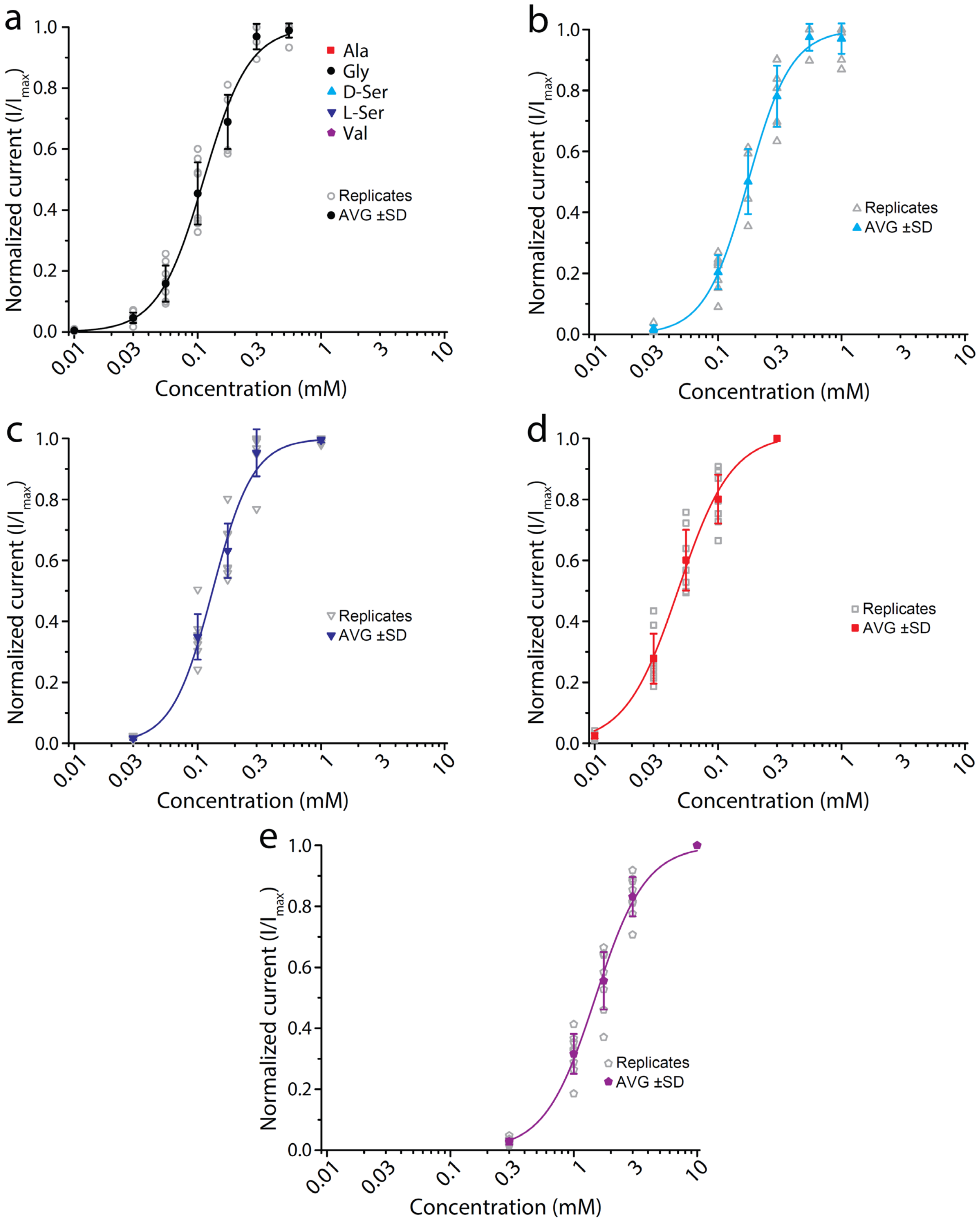

Supplementary Figure 5: Separate dose response curves for activating ligands of GluE1αA. a Glycine dose response curve (n=8). **b** D-serine dose response curve (n=7). **c** L-serine dose response curve (n=7). **d** Alanine dose response curve (n=7). **e** Valine dose response curve (n=7).

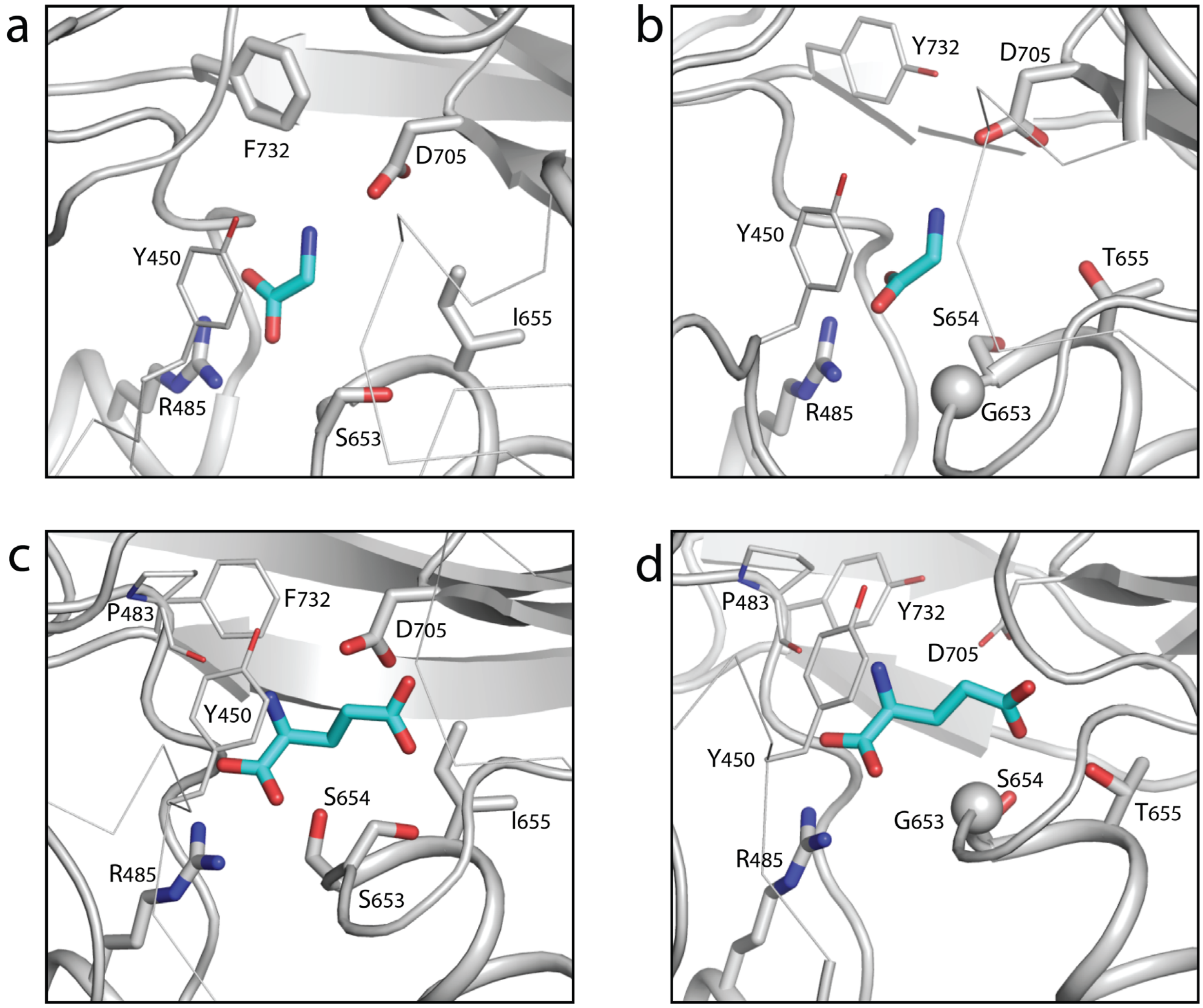

Supplementary Figure 6: Alternate depiction of docked glycine and glutamate ligands in the ligand binding pocket of wildtype and m3 mutant GluE1αA. **a** Homology modeling and docking of glycine in the putative ligand binding pocket of the wiltype GluE1αA receptor. **b** Homology modeling and docking of glycine in triple mutant (m3) variant of GluE1αA. **c** Homology modeling and docking of glutamate in the wt GluE1αA receptor. **d** Homology modeling and docking of glutamate in triple mutant (m3) variant of GluE1αA.

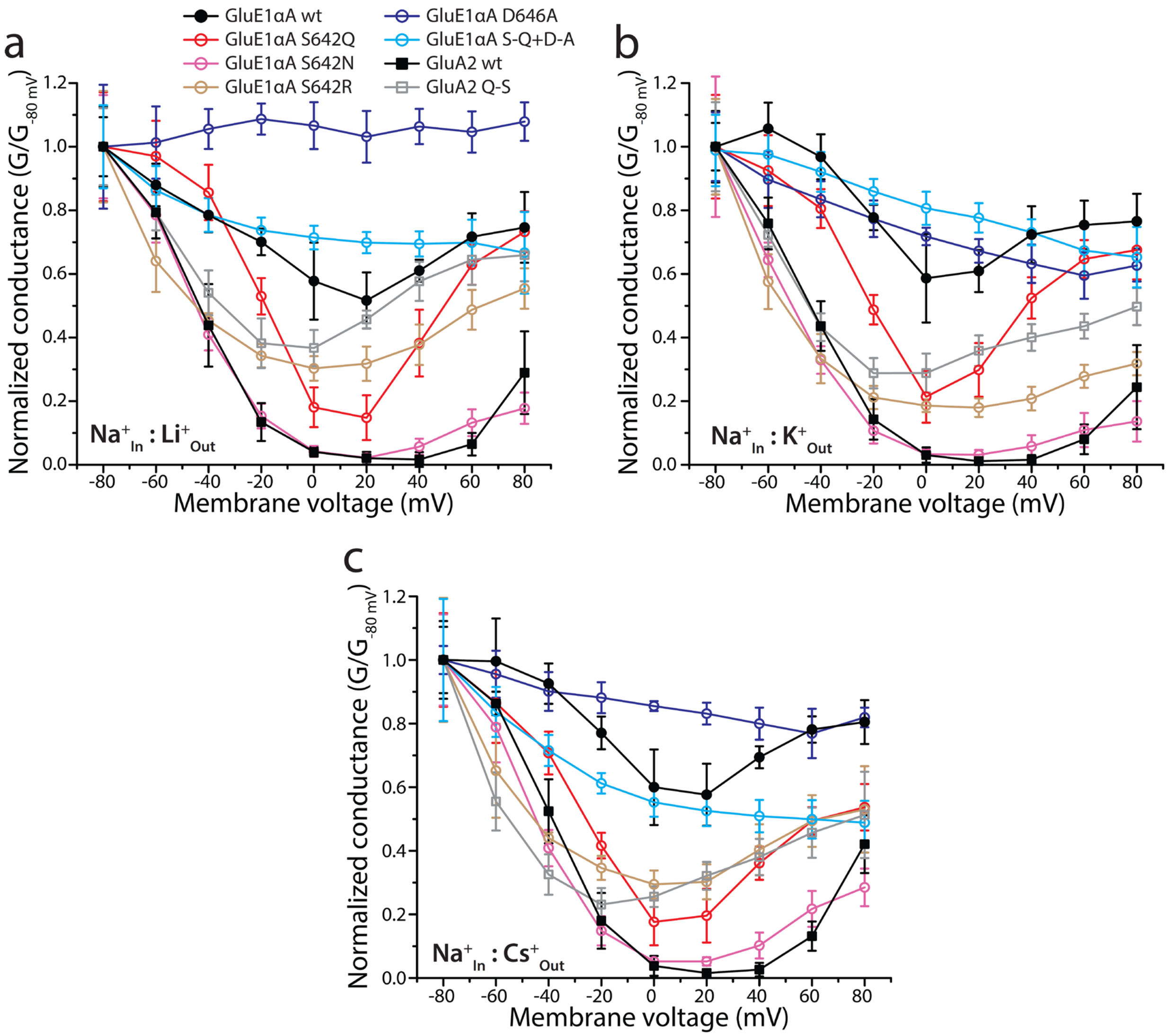

Supplementary Figure 7: Average normalized conductance vs. membrane voltage (G-V) plots for the remaining bi-ionic conditions presented in Fig. 5. a Plot of average normalized G-V data recorded using equimolar $[Li^+]_{out}$ and $[Na^+]_{in}$ for the different variants of GluE1αA and human GluA2 receptors. **b** G-V plot for equimolar $[K^+]_{out}$ and $[Na^+]_{in}$. **c** G-V plot for equimolar $[Cs^+]_{out}$ and $[Na^+]_{in}$. The legend embedded in panel a pertains to all panels.

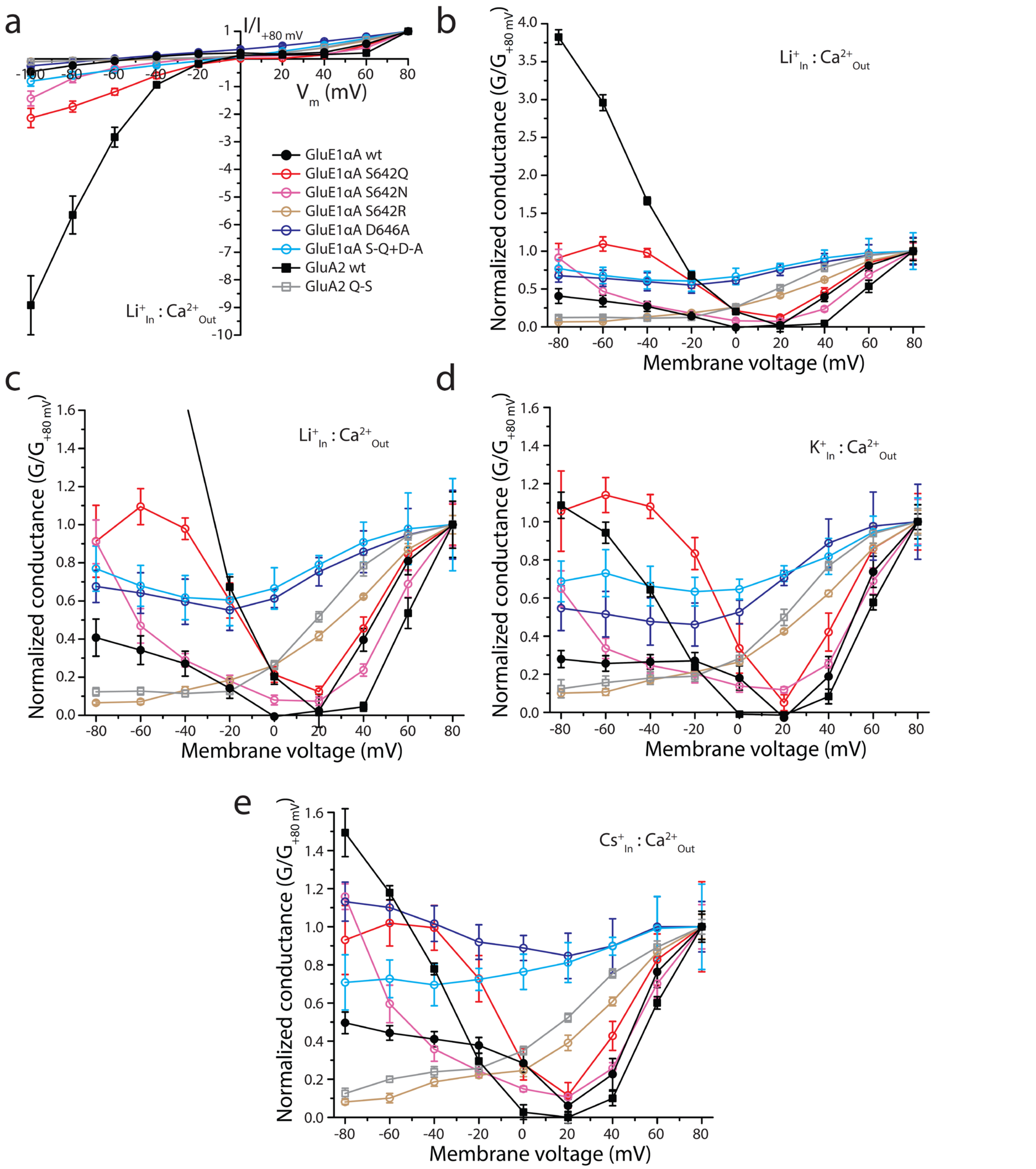

Supplementary Figure 8: Expanded I-V plot for Li^+ vs. Ca^{2+} and average normalized G-V plots for the remaining bi-ionic conditions presented in Fig. 6. a Plot of average normalized current vs. membrane voltages (I-V) under 4 mM extracellular Ca^{2+} and 100 mM intracellular Li^+ conducted by wildtype and pore mutant variants of GluE1αA, the wildtype human GluA2 receptor, and a mutant GluA2 variant bearing a glutamine to serine mutation in the QRN site (n=7-8). **b** Plot of average normalized G-V data recorded using 4 mM $[Ca^{2+}]_{out}$ and 100 mM $[Li^+]_{in}$ for the different variants of GluE1αA and human GluA2 receptors. **c** Scaled plot of average normalized G-V data for the plot in panel b. **d** G-V plot for 4 mM $[Ca^{2+}]_{out}$ and 100 mM $[K^+]_{in}$. **e** G-V plot for 4 mM $[Ca^{2+}]_{out}$ and 100 mM $[Cs^+]_{in}$. The legend embedded in panel a pertains to all panels.

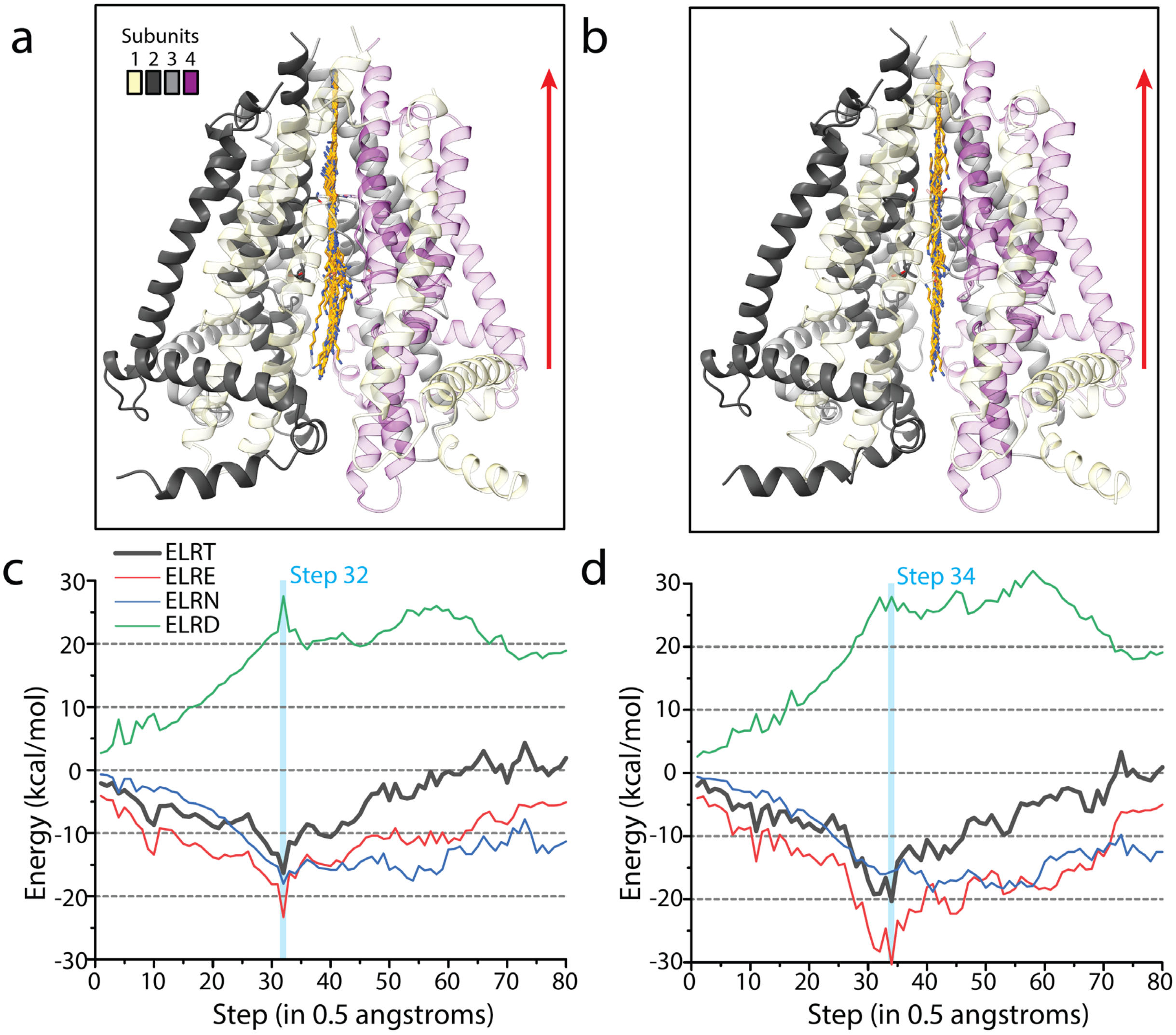

Supplementary Figure 9. Spermine in the pore of GluE1αA. a Superposition of 80 MC-minimized structures of spermine within the wildtype receptor (only a single MC-minimized structure of the receptor is shown for clarity). **b** Similar superposition of 80 MC-minimized spermine structures in the N_{642}N variant. For panels a and b, side chains of Q/R/N and +4 residues are shown as sticks. Spermine is shown in orange carbons and colored heteroatoms. The front subunits 1 and 4 of the receptors are semi-transparent for clarity. The red arrows show the direction in which spermine was pulled. **c** Plot of Monte Carlo-minimized energy as spermine was pulled through the open wildtype GluE1αA pore, from the cytoplasmic to the extracellular side, in 0.5 Å steps. **d** Plot of Monte Carlo-minimized energy as spermine was pulled through the open the S_{642}N variant pore. For panels c to d, ELRT: Energy Ligand Receptor - Total; ELRE: Energy Ligand Receptor - Electrostatic; ELRN: Energy Ligand Receptor - Non-bonded; ELRD: Energy Ligand Receptor - Desolvation.

Supplementary Table 1. Details about proteomes used for the species aware gene tree of iGluR protein sequences.

Clade	Clade	Clade	Clade	Clade	Clade	Clade	Clade	Clade	Species	Source	BUSCO (Proteome vs eukaryota_odb10)
Amorphea	Amoebozoa	Discosea							Acanthamoeba castellanii	https://figshare.com/articles/dataset/EukProt_a_datab	C:199 [S:195, D:4], F:15, M:41, n:255
Amorphea	Amoebozoa	Discosea							Dracoamoeba jomungandri	https://figshare.com/articles/dataset/EukProt_a_datab	C:169 [S:167, D:2], F:25, M:61, n:255
Amorphea	Amoebozoa	Evosea							Dictyostelium discoideum	https://figshare.com/articles/dataset/EukProt_a_datab	C:240 [S:230, D:10], F:3, M:12, n:255
Amorphea	Amoebozoa	Evosea							Filamoeba spATCC50430	https://figshare.com/articles/dataset/EukProt_a_datab	C:182 [S:120, D:62], F:17, M:56, n:255
Amorphea	Amoebozoa	Evosea							Mastigamoeba balamuthi	https://figshare.com/articles/dataset/EukProt_a_datab	C:204 [S:198, D:6], F:11, M:40, n:255
Amorphea	Amoebozoa	Tubulinea							Amoeba proteus	https://figshare.com/articles/dataset/EukProt_a_datab	C:192 [S:61, D:131], F:24, M:39, n:255
Amorphea	Amoebozoa	Tubulinea							Arcella intermedia	https://figshare.com/articles/dataset/EukProt_a_datab	C:216 [S:181, D:35], F:4, M:35, n:255
Amorphea	Amoebozoa	Tubulinea							Vermamoeba vermiformis	https://figshare.com/articles/dataset/EukProt_a_datab	C:228 [S:215, D:13], F:8, M:19, n:255
Amorphea	Obazoa	Apusomonadida							Apusomonadida spAF17	https://figshare.com/articles/dataset/EukProt_a_datab	C:106 [S:105, D:1], F:48, M:101, n:255
Amorphea	Obazoa	Apusomonadida							Thecamonas trahens	https://figshare.com/articles/dataset/EukProt_a_datab	C:211 [S:211, D:0], F:15, M:29, n:255
Amorphea	Obazoa	Breviatea							Lenisia limosa	https://figshare.com/articles/dataset/EukProt_a_datab	C:118 [S:110, D:8], F:47, M:90, n:255
Amorphea	Obazoa	Breviatea							Pygsuia biforma	https://figshare.com/articles/dataset/EukProt_a_datab	C:159 [S:159, D:0], F:38, M:58, n:255
Amorphea	Obazoa	Opisthokonta	Holozoa						Tunicaraptor unikontum	https://www.ncbi.nlm.nih.gov/search/all/?term=PRJNA	C:191 [S:167, D:24], F:40, M:24, n:255
Amorphea	Obazoa	Opisthokonta	Holozoa	Choanoflagellate					Codosiga hollandica	https://figshare.com/articles/dataset/EukProt_a_datab	C:226 [S:196, D:30], F:15, M:14, n:255
Amorphea	Obazoa	Opisthokonta	Holozoa	Choanoflagellate					Diaphanoeca grandis	https://figshare.com/articles/dataset/EukProt_a_datab	C:235 [S:230, D:5], F:8, M:12, n:255
Amorphea	Obazoa	Opisthokonta	Holozoa	Choanoflagellate					Monosiga brevicollis	https://figshare.com/articles/dataset/EukProt_a_datab	C:203 [S:203, D:0], F:17, M:35, n:255
Amorphea	Obazoa	Opisthokonta	Holozoa	Choanoflagellate					Salpingoeca dolichothecata	https://figshare.com/articles/dataset/EukProt_a_datab	C:233 [S:227, D:6], F:13, M:9, n:255
Amorphea	Obazoa	Opisthokonta	Holozoa	Choanoflagellate					Salpingoeca kvevrii	https://figshare.com/articles/dataset/EukProt_a_datab	C:243 [S:223, D:20], F:7, M:5, n:255
Amorphea	Obazoa	Opisthokonta	Holozoa	Choanoflagellate					Salpingoeca rosetta	https://figshare.com/articles/dataset/EukProt_a_datab	C:212 [S:211, D:1], F:17, M:26, n:255
Amorphea	Obazoa	Opisthokonta	Holozoa	Filasterea					Capsaspora owczarzaki	https://figshare.com/articles/dataset/EukProt_a_datab	C:236 [S:234, D:2], F:8, M:11, n:255
Amorphea	Obazoa	Opisthokonta	Holozoa	Ichthyosporea					Amoebidium parasiticum	https://figshare.com/articles/dataset/EukProt_a_datab	C:230 [S:211, D:19], F:16, M:9, n:255
Amorphea	Obazoa	Opisthokonta	Holozoa	Ichthyosporea					Creolimax fragrantissima	https://figshare.com/articles/dataset/EukProt_a_datab	C:244 [S:240, D:4], F:5, M:6, n:255
Amorphea	Obazoa	Opisthokonta	Holozoa	Ichthyosporea					Ichthyophonus hoferi	https://figshare.com/articles/dataset/EukProt_a_datab	C:137 [S:136, D:1], F:34, M:84, n:255
Amorphea	Obazoa	Opisthokonta	Holozoa	Ichthyosporea					Sphaeroforma arctica	https://figshare.com/articles/dataset/EukProt_a_datab	C:162 [S:154, D:8], F:59, M:34, n:255
Amorphea	Obazoa	Opisthokonta	Holozoa	Ichthyosporea					Sphaerothecum destruens	https://figshare.com/articles/dataset/EukProt_a_datab	C:176 [S:141, D:35], F:60, M:19, n:255
Amorphea	Obazoa	Opisthokonta	Holozoa	Ichthyosporea					Syssomonas multiformis	https://figshare.com/articles/dataset/EukProt_a_datab	C:148 [S:95, D:53], F:37, M:70, n:255
Amorphea	Obazoa	Opisthokonta	Holozoa	Metazoa	Bilateria	Deuterostome	Ambulacraria	Echinoderm	Acanthaster planci	https://marinegenomics.oist.jp/cots/viewer/download?	C:234 [S:234, D:0], F:14, M:7, n:255
Amorphea	Obazoa	Opisthokonta	Holozoa	Metazoa	Bilateria	Deuterostome	Ambulacraria	Echinoderm	Anneissia japonica	https://www.ncbi.nlm.nih.gov/search/all/?term=GCA_(	C:246 [S:184, D:62], F:4, M:5, n:255
Amorphea	Obazoa	Opisthokonta	Holozoa	Metazoa	Bilateria	Deuterostome	Ambulacraria	Echinoderm	Strongylocentrotus purpuratus	https://figshare.com/articles/dataset/EukProt_a_datab	C:245 [S:173, D:72], F:8, M:2, n:255
Amorphea	Obazoa	Opisthokonta	Holozoa	Metazoa	Bilateria	Deuterostome	Ambulacraria	Hemichordata	Ptychodera flava	https://groups.oist.jp/molgenu/hemichordate-genome:	C:137 [S:133, D:4], F:62, M:56, n:255
Amorphea	Obazoa	Opisthokonta	Holozoa	Metazoa	Bilateria	Deuterostome	Ambulacraria	Hemichordata	Saccoglossus kowalevskii	https://groups.oist.jp/molgenu/hemichordate-genome:	C:240 [S:233, D:7], F:9, M:6, n:255
Amorphea	Obazoa	Opisthokonta	Holozoa	Metazoa	Bilateria	Deuterostome	Chordate	Cephalochordate	Branchiostoma belcheri	https://www.ncbi.nlm.nih.gov/datasets/genome/GCF_(	C:254 [S:214, D:40], F:0, M:1, n:255
Amorphea	Obazoa	Opisthokonta	Holozoa	Metazoa	Bilateria	Deuterostome	Chordate	Cephalochordate	Branchiostoma floridae	https://figshare.com/articles/dataset/EukProt_a_datab	C:225 [S:221, D:4], F:20, M:10, n:255
Amorphea	Obazoa	Opisthokonta	Holozoa	Metazoa	Bilateria	Deuterostome	Chordate	Urochordate	Ciona intestinalis	https://figshare.com/articles/dataset/EukProt_a_datab	C:248 [S:218, D:30], F:4, M:3, n:255
Amorphea	Obazoa	Opisthokonta	Holozoa	Metazoa	Bilateria	Deuterostome	Chordate	Vertebrate	Danio rerio	https://figshare.com/articles/dataset/EukProt_a_datab	C:255 [S:194, D:61], F:0, M:0, n:255
Amorphea	Obazoa	Opisthokonta	Holozoa	Metazoa	Bilateria	Deuterostome	Chordate	Vertebrate	Eptatretus burgeri	https://ftp.ensembl.org/pub/release-111/fasta/eptatre	C:220 [S:117, D:103], F:28, M:7, n:255
Amorphea	Obazoa	Opisthokonta	Holozoa	Metazoa	Bilateria	Deuterostome	Chordate	Vertebrate	Gallus gallus	https://figshare.com/articles/dataset/EukProt_a_datab	C:254 [S:163, D:91], F:0, M:1, n:255
Amorphea	Obazoa	Opisthokonta	Holozoa	Metazoa	Bilateria	Deuterostome	Chordate	Vertebrate	Homo sapiens	https://figshare.com/articles/dataset/EukProt_a_datab	C:255 [S:96, D:159], F:0, M:0, n:255
Amorphea	Obazoa	Opisthokonta	Holozoa	Metazoa	Bilateria	Deuterostome	Chordate	Vertebrate	Petromyzon marinus	https://www.ncbi.nlm.nih.gov/datasets/taxonomy/775	C:241 [S:151, D:90], F:7, M:7, n:255
Amorphea	Obazoa	Opisthokonta	Holozoa	Metazoa	Bilateria	Deuterostome	Chordate	Vertebrate	Rattus norvegicus	https://www.ncbi.nlm.nih.gov/search/all/?term=GCF_0	C:253 [S:116, D:137], F:1, M:1, n:255
Amorphea	Obazoa	Opisthokonta	Holozoa	Metazoa	Bilateria	Protostome	Ecdysozoa	Arthropod	Calanus glacialis	https://figshare.com/articles/dataset/EukProt_a_datab	C:247 [S:214, D:33], F:6, M:2, n:255
Amorphea	Obazoa	Opisthokonta	Holozoa	Metazoa	Bilateria	Protostome	Ecdysozoa	Arthropod	Drosophila melanogaster	https://figshare.com/articles/dataset/EukProt_a_datab	C:255 [S:154, D:101], F:0, M:0, n:255
Amorphea	Obazoa	Opisthokonta	Holozoa	Metazoa	Bilateria	Protostome	Ecdysozoa	Arthropod	Strigamia marittima	http://ftp.ensemblgenomes.org/pub/metazoa/release-!	C:236 [S:217, D:19], F:6, M:13, n:255
Amorphea	Obazoa	Opisthokonta	Holozoa	Metazoa	Bilateria	Protostome	Ecdysozoa	Nematode	Caenorhabditis elegans	https://figshare.com/articles/dataset/EukProt_a_datab	C:250 [S:222, D:28], F:2, M:3, n:255
Amorphea	Obazoa	Opisthokonta	Holozoa	Metazoa	Bilateria	Protostome	Ecdysozoa	Nematode	Loa loa	https://www.ncbi.nlm.nih.gov/search/all/?term=GCF_0	C:242 [S:230, D:12], F:5, M:8, n:255
Amorphea	Obazoa	Opisthokonta	Holozoa	Metazoa	Bilateria	Protostome	Ecdysozoa	Tardigrad	Ramazzottius varieornatus	https://www.uniprot.org/proteomes/UP000186922	C:237 [S:226, D:11], F:7, M:11, n:255
Amorphea	Obazoa	Opisthokonta	Holozoa	Metazoa	Bilateria	Protostome	Spiralia	Annelida	Capitella teleta	https://figshare.com/articles/dataset/EukProt_a_datab	C:240 [S:235, D:5], F:11, M:4, n:255
Amorphea	Obazoa	Opisthokonta	Holozoa	Metazoa	Bilateria	Protostome	Spiralia	Annelida	Helobdella robusta	https://www.ncbi.nlm.nih.gov/search/all/?term=GCF_0	C:248 [S:246, D:2], F:4, M:3, n:255
Amorphea	Obazoa	Opisthokonta	Holozoa	Metazoa	Bilateria	Protostome	Spiralia	Brachiopoda	Lingula unguis	https://www.uniprot.org/proteomes/UP000085678	C:249 [S:197, D:52], F:3, M:3, n:255
Amorphea	Obazoa	Opisthokonta	Holozoa	Metazoa	Bilateria	Protostome	Spiralia	Mollusca	Crassostrea gigas	https://figshare.com/articles/dataset/EukProt_a_datab	C:252 [S:192, D:60], F:3, M:0, n:255
Amorphea	Obazoa	Opisthokonta	Holozoa	Metazoa	Bilateria	Protostome	Spiralia	Mollusca	Octopus bimaculoides	https://figshare.com/articles/dataset/EukProt_a_datab	C:237 [S:188, D:49], F:15, M:3, n:255
Amorphea	Obazoa	Opisthokonta	Holozoa	Metazoa	Cnidaria	Anthozoa	Hexacorallia	Actiniaria	Nematostella vectensis	https://figshare.com/articles/dataset/EukProt_a_datab	C:239 [S:234, D:5], F:13, M:3, n:255
Amorphea	Obazoa	Opisthokonta	Holozoa	Metazoa	Cnidaria	Anthozoa	Hexacorallia	Scleractinia	Fungia scutaria (Lobactis scutaria)	http://comparative.reefgenomics.org/datasets.html	C:216 [S:211, D:5], F:35, M:4, n:255
Amorphea	Obazoa	Opisthokonta	Holozoa	Metazoa	Cnidaria	Anthozoa	Hexacorallia	Scleractinia	Stylophora pistillata	https://www.uniprot.org/proteomes/UP000225706	C:217 [S:216, D:1], F:23, M:15, n:255
Amorphea	Obazoa	Opisthokonta	Holozoa	Metazoa	Cnidaria	Cubozoa			Alatina alata	https://bitbucket.org/caseywdunn/cnidaria2014-assem	C:246 [S:195, D:51], F:6, M:3, n:255
Amorphea	Obazoa	Opisthokonta	Holozoa	Metazoa	Cnidaria	Hydrozoa			Clytia hemisphaerica	http://marimba.obs-vlfr.fr/organism/Clytia/hemisphaer	C:236 [S:146, D:90], F:12, M:7, n:255
Amorphea	Obazoa	Opisthokonta	Holozoa	Metazoa	Cnidaria	Hydrozoa			Hydra vulgaris	https://research.nhgri.nih.gov/hydra/	C:224 [S:214, D:10], F:23, M:8, n:255
Amorphea	Obazoa	Opisthokonta	Holozoa	Metazoa	Cnidaria	Scyphozoa			Aurelia aurita	https://davidadlergold.faculty.ucdavis.edu/jellyfish/	C:172 [S:152, D:20], F:24, M:59, n:255
Amorphea	Obazoa	Opisthokonta	Holozoa	Metazoa	Cnidaria	Scyphozoa			Rhopilema esculentum	https://academic.oup.com/gigascience/article/9/4/giaa	C:250 [S:159, D:91], F:3, M:2, n:255
Amorphea	Obazoa	Opisthokonta	Holozoa	Metazoa	Ctenophora				Hormiphora californiensis	Adriano Senatore Lab	C:242 [S:101, D:141], F:4, M:9, n:255
Amorphea	Obazoa	Opisthokonta	Holozoa	Metazoa	Ctenophora				Mnemiopsis leidyi	https://figshare.com/articles/dataset/EukProt_a_datab	C:213 [S:210, D:3], F:20, M:22, n:255
Amorphea	Obazoa	Opisthokonta	Holozoa	Metazoa	Placozoa				Hoilungia hongkongensis	https://github.com/xgrau/placozoa-cell-type-evolution-	C:237 [S:227, D:10], F:6, M:12, n:255
Amorphea	Obazoa	Opisthokonta	Holozoa	Metazoa	Placozoa				Hoilungia sp H23	https://github.com/xgrau/placozoa-cell-type-evolution-	C:249 [S:249, D:0], F:4, M:2, n:255
Amorphea	Obazoa	Opisthokonta	Holozoa	Metazoa	Placozoa				Trichoplax adhaerens	Adriano Senatore Lab	C:253 [S:252, D:1], F:1, M:1, n:255
Amorphea	Obazoa	Opisthokonta	Holozoa	Metazoa	Placozoa				Trichoplax sp H2	https://figshare.com/articles/dataset/EukProt_a_datab	C:245 [S:242, D:3], F:6, M:4, n:255
Amorphea	Obazoa	Opisthokonta	Holozoa	Metazoa	Porifera	Calcarea			Leucosolenia complicata	https://compagen.unit.oist.jp/datasets.html	C:242 [S:204, D:38], F:7, M:6, n:255
Amorphea	Obazoa	Opisthokonta	Holozoa	Metazoa	Porifera	Calcarea			Sycon ciliatum	https://compagen.unit.oist.jp/datasets.html	C:248 [S:232, D:16], F:5, M:2, n:255
Amorphea	Obazoa	Opisthokonta	Holozoa	Metazoa	Porifera	Demospongiae			Amphimedon queenslandica	https://figshare.com/articles/dataset/EukProt_a_datab	C:243 [S:199, D:44], F:7, M:5, n:255
Amorphea	Obazoa	Opisthokonta	Holozoa	Metazoa	Porifera	Demospongiae			Ephydatia muelleri	https://spaces.facsci.ualberta.ca/ephybase/	C:248 [S:207, D:41], F:4, M:3, n:255
Amorphea	Obazoa	Opisthokonta	Holozoa	Metazoa	Porifera	Demospongiae			Haliclona tubifera	https://compagen.unit.oist.jp/datasets.html	C:192 [S:189, D:3], F:8, M:55, n:255
Amorphea	Obazoa	Opisthokonta	Holozoa	Metazoa	Porifera	Homoscleromorpha			Oscarella carmela	https://compagen.unit.oist.jp/datasets.html	C:117 [S:115, D:2], F:55, M:83, n:255
Amorphea	Obazoa	Opisthokonta	Holozoa	Metazoa	Porifera	Homoscleromorpha			Oscarella pearsei	https://figshare.com/articles/dataset/EukProt_a_datab	C:242 [S:237, D:5], F:6, M:7, n:255
Amorphea	Obazoa	Opisthokonta	Nucletmycea(/Holc	Fungi	Blastocladiomycota				Allomyces macrogynus	https://figshare.com/articles/dataset/EukProt_a_datab	C:237 [S:61, D:176], F:7, M:11, n:255
Amorphea	Obazoa	Opisthokonta	Nucletmycea(/Holc	Fungi	Ascomycota				Aspergillus nidulans	https://figshare.com/articles/dataset/EukProt_a_datab	C:227 [S:225, D:2], F:16, M:12, n:255
Amorphea	Obazoa	Opisthokonta	Nucletmycea(/Holc	Fungi	Chytridiomycota				Batrachochytrium dendrobatidis	https://figshare.com/articles/dataset/EukProt_a_datab	C:236 [S:232, D:4], F:12, M:7, n:255
Amorphea	Obazoa	Opisthokonta	Nucletmycea(/Holc	Fungi	Entomophthoromycota				Conidiobolus coronatus	https://figshare.com/articles/dataset/EukProt_a_datab	C:206 [S:203, D:3], F:11, M:38, n:255
Amorphea	Obazoa	Opisthokonta	Nucletmycea(/Holc	Fungi	Basidiomycota				Cryptococcus neoformans	https://figshare.com/articles/dataset/EukProt_a_datab	C:246 [S:238, D:8], F:6, M:3, n:255
Amorphea	Obazoa	Opisthokonta	Nucletmycea(/Holc	Fungi	Ascomycota				Neurospora crassa	https://figshare.com/articles/dataset/EukProt_a_datab	C:255 [S:237, D:18], F:0, M:0, n:255
Amorphea	Obazoa	Opisthokonta	Nucletmycea(/Holc	Fungi	Aphelidiomycota				Paraphelidium tribonemae	https://figshare.com/articles/dataset/EukProt_a_datab	C:226 [S:125, D:101], F:7, M:22, n:255
Amorphea	Obazoa	Opisthokonta	Nucletmycea(/Holc	Fungi	Glomeromycota				Rhizophagus irregularis	https://figshare.com/articles/dataset/EukProt_a_datab	C:242 [S:239, D:3], F:5, M:8, n:255
Amorphea	Obazoa	Opisthokonta	Nucletmycea(/Holc	Fungi	Zygomycota				Rhizopus delemar	https://figshare.com/articles/dataset/EukProt_a_datab	C:191 [S:165, D:26], F:44, M:20, n:255
Amorphea	Obazoa	Opisthokonta	Nucletmycea(/Holc	Fungi	Blastocladiomycota				Rozella allomycis	https://figshare.com/articles/dataset/EukProt_a_datab	C:184 [S:184, D:0], F:27, M:44, n:255
Amorphea	Obazoa	Opisthokonta	Nucletmycea(/Holc	Fungi	Ascomycota				Saccharomyces cerevisiae	https://figshare.com/articles/dataset/EukProt_a_datab	C:239 [S:237, D:2], F:2, M:14, n:255
Amorphea	Obazoa	Opisthokonta	Nucletmycea(/Holc	Fungi	Ascomycota				Schizosaccharomyces pombe	https://figshare.com/articles/dataset/EukProt_a_datab	C:247 [S:243, D:4], F:1, M:7, n:255
Amorphea	Obazoa	Opisthokonta	Nucletmycea(/Holc	Fungi	Basidiomycota				Ustilago maydis	https://figshare.com/articles/dataset/EukProt_a_datab	C:253 [S:253, D:0], F:0, M:2, n:255
Amorphea	Obazoa	Opisthokonta	Nucletmycea(/Holc	Fungi	Ascomycota				Yarrowia lipolytica	https://figshare.com/articles/dataset/EukProt_a_datab	C:242 [S:242, D:0], F:8, M:5, n:255
Amorphea	Obazoa	Opisthokonta	Nucletmycea(/Holc	Rotosphaerida					Fonticula alba	https://figshare.com/articles/dataset/EukProt_a_datab	C:183 [S:176, D:7], F:25, M:47, n:255
Amorphea	Obazoa	Opisthokonta	Nucletmycea(/Holc	Rotosphaerida					Parvularia atlantis	https://figshare.com/articles/dataset/EukProt_a_datab	C:194 [S:181, D:13], F:47, M:14, n:255
Archaeplastida	Chloroplastida	Viridiplantae	Chlorophyta						Chlamydomonas reinhardtii	https://figshare.com/articles/dataset/EukProt_a_datab	C:197 [S:197, D:0], F:30, M:28, n:255
Archaeplastida	Chloroplastida	Viridiplantae	Chlorophyta						Chlorella variabilis	https://figshare.com/articles/dataset/EukProt_a_datab	C:202 [S:201, D:1], F:18, M:35, n:255
Archaeplastida	Chloroplastida	Viridiplantae	Chlorophyta						Micromonas pusilla	https://figshare.com/articles/dataset/EukProt_a_datab	C:206 [S:204, D:2], F:13, M:36, n:255
Archaeplastida	Chloroplastida	Viridiplantae	Chlorophyta						Nephroselmis pyriformis	https://figshare.com/articles/dataset/EukProt_a_datab	C:166 [S:163, D:3], F:56, M:33, n:255
Archaeplastida	Chloroplastida	Viridiplantae	Chlorophyta						Ostreococcus tauri	https://figshare.com/articles/dataset/EukProt_a_datab	C:207 [S:206, D:1], F:13, M:35, n:255
Archaeplastida	Chloroplastida	Viridiplantae	Chlorophyta						Volvox carteri	https://figshare.com/articles/dataset/EukProt_a_datab	C:205 [S:204, D:1], F:20, M:30, n:255
Archaeplastida	Chloroplastida	Viridiplantae	Klebsormidiophyceae						Klebsormidium nitens	https://figshare.com/articles/dataset/EukProt_a_datab	C:249 [S:248, D:1], F:3, M:3, n:255
Archaeplastida	Chloroplastida	Viridiplantae	Prasinodermophyta						Prasinoderma coloniale	https://figshare.com/articles/dataset/EukProt_a_datab	C:217 [S:217, D:0], F:13, M:25, n:255
Archaeplastida	Chloroplastida	Viridiplantae	Streptophyta						Amborella trichopoda	https://figshare.com/articles/dataset/EukProt_a_datab	C:253 [S:115, D:138], F:2, M:0, n:255
Archaeplastida	Chloroplastida	Viridiplantae	Streptophyta						Arabidopsis thaliana	https://figshare.com/articles/dataset/EukProt_a_datab	C:255 [S:109, D:146], F:0, M:0, n:255
Archaeplastida	Chloroplastida	Viridiplantae	Streptophyta						Marchantia polymorpha	https://figshare.com/articles/dataset/EukProt_a_datab	C:255 [S:212, D:43], F:0, M:0, n:255
Archaeplastida	Chloroplastida	Viridiplantae	Streptophyta						Physcomitrium patens	https://figshare.com/articles/dataset/EukProt_a_datab	C:255 [S:116, D:139], F:0, M:0, n:255
Archaeplastida	Chloroplastida	Viridiplantae	Streptophyta						Populus trichocarpa	https://figshare.com/articles/dataset/EukProt_a_datab	C:254 [S:86, D:168], F:0, M:1, n:255
Archaeplastida	Chloroplastida	Viridiplantae	Streptophyta						Selaginella moellendorffii	https://figshare.com/articles/dataset/EukProt_a_datab	C:253 [S:6, D:247], F:1, M:1, n:255
Archaeplastida	Glaucophyta								Cyanophora paradoxa	https://figshare.com/articles/dataset/EukProt_a_datab	C:218 [S:212, D:6], F:9, M:28, n:255
Archaeplastida	Glaucophyta								Gloeochaete wittrockiana	https://figshare.com/articles/dataset/EukProt_a_datab	C:221 [S:164, D:57], F:12, M:22, n:255
Archaeplastida	Rhodophyta								Bangiopsis sp CCMP1999	https://figshare.com/articles/dataset/EukProt_a_datab	C:146 [S:145, D:1], F:20, M:89, n:255
Archaeplastida	Rhodophyta								Chondrus crispus	https://figshare.com/articles/dataset/EukProt_a_datab	C:164 [S:159, D:5], F:18, M:73, n:255
Archaeplastida	Rhodophyta								Cyanidioschyzon merolae	https://figshare.com/articles/dataset/EukProt_a_datab	C:188 [S:185, D:3], F:14, M:53, n:255
Archaeplastida	Rhodophyta								Eucheuma denticulatum	https://figshare.com/articles/dataset/EukProt_a_datab	C:128 [S:115, D:13], F:23, M:104, n:255
Archaeplastida	Rhodophyta								Galdieria sulphuraria	https://figshare.com/articles/dataset/EukProt_a_datab	C:204 [S:177, D:27], F:15, M:36, n:255
Archaeplastida	Rhodophyta								Gracilariopsis chorda	https://figshare.com/articles/dataset/EukProt_a_datab	C:208 [S:203, D:5], F:11, M:36, n:255
Archaeplastida	Rhodophyta								Rhodelphis limneticus	https://figshare.com/articles/dataset/EukProt_a_datab	C:171 [S:169, D:2], F:42, M:42, n:255
Archaeplastida	Rhodophyta								Rhodelphis marinus	https://figshare.com/articles/dataset/EukProt_a_datab	C:229 [S:227, D:2], F:13, M:13, n:255
Archaeplastida	Rhodophyta								Rhodosorus marinus	https://figshare.com/articles/dataset/EukProt_a_datab	C:187 [S:130, D:57], F:14, M:54, n:255
CRuMS	Collodictyonidae								Diphylleia rotans	https://figshare.com/articles/dataset/EukProt_a_datab	C:231 [S:139, D:92], F:6, M:18, n:255
CRuMS	Rigifilida								Rigifila ramosa	https://figshare.com/articles/dataset/EukProt_a_datab	C:221 [S:169, D:52], F:17, M:17, n:255
Cryptista	Cryptophyceae								Baffinella frigidus	https://figshare.com/articles/dataset/EukProt_a_datab	C:205 [S:174, D:31], F:25, M:25, n:255
Cryptista	Cryptophyceae								Cryptomonas curvata	https://figshare.com/articles/dataset/EukProt_a_datab	C:107 [S:89, D:18], F:39, M:109, n:255
Cryptista	Cryptophyceae								Geminigera cryophila	https://figshare.com/articles/dataset/EukProt_a_datab	C:186 [S:160, D:26], F:24, M:45, n:255
Cryptista	Cryptophyceae								Goniomonas avonlea	https://figshare.com/articles/dataset/EukProt_a_datab	C:111 [S:98, D:13], F:25, M:119, n:255
Cryptista	Cryptophyceae								Goniomonas pacifica	https://figshare.com/articles/dataset/EukProt_a_datab	C:178 [S:161, D:17], F:40, M:37, n:255
Cryptista	Cryptophyceae								Guillardia theta	https://figshare.com/articles/dataset/EukProt_a_datab	C:204 [S:195, D:9], F:25, M:26, n:255
Cryptista									Palpitomonas bilix	https://figshare.com/articles/dataset/EukProt_a_datab	C:151 [S:69, D:82], F:31, M:73, n:255
Discoba	Euglenozoa								Bodo saltans	https://figshare.com/articles/dataset/EukProt_a_datab	C:141 [S:137, D:4], F:23, M:91, n:255
Discoba	Euglenozoa								Entosiphon sulcatum	https://figshare.com/articles/dataset/EukProt_a_datab	C:178 [S:148, D:30], F:30, M:47, n:255
Discoba	Euglenozoa								Euglena gracilis	https://figshare.com/articles/dataset/EukProt_a_datab	C:185 [S:177, D:8], F:34, M:36, n:255
Discoba	Euglenozoa								Euglena longa	https://figshare.com/articles/dataset/EukProt_a_datab	C:214 [S:182, D:32], F:16, M:25, n:255
Discoba	Euglenozoa								Hemistasia phaeocysticola	https://figshare.com/articles/dataset/EukProt_a_datab	C:174 [S:145, D:29], F:22, M:59, n:255
Discoba	Euglenozoa								Leishmania major	https://figshare.com/articles/dataset/EukProt_a_datab	C:137 [S:131, D:6], F:25, M:93, n:255
Discoba	Euglenozoa								Rhynchopus humris	https://figshare.com/articles/dataset/EukProt_a_datab	C:176 [S:127, D:49], F:19, M:60, n:255
Discoba	Euglenozoa								Sulcionema specki	https://figshare.com/articles/dataset/EukProt_a_datab	C:160 [S:157, D:3], F:39, M:56, n:255
Discoba	Euglenozoa								Trypanosoma brucei	https://figshare.com/articles/dataset/EukProt_a_datab	C:144 [S:139, D:5], F:16, M:95, n:255
Discoba	Heterolobosea								Naegleria gruberi	https://figshare.com/articles/dataset/EukProt_a_datab	C:204 [S:199, D:5], F:20, M:31, n:255
Discoba	Heterolobosea								Neovahlkampfia damariscottae	https://figshare.com/articles/dataset/EukProt_a_datab	C:221 [S:217, D:4], F:12, M:22, n:255
Discoba	Heterolobosea								Pharyngomonas kirbyi	https://figshare.com/articles/dataset/EukProt_a_datab	C:217 [S:214, D:3], F:17, M:21, n:255
Discoba	Jakobida								Andalucia godoyi	https://figshare.com/articles/dataset/EukProt_a_datab	C:203 [S:202, D:1], F:13, M:39, n:255
Discoba	Jakobida								Stygiella incarcerata	https://figshare.com/articles/dataset/EukProt_a_datab	C:176 [S:127, D:49], F:15, M:64, n:255
Haptista	Centroplasthelida								Acanthocystis sp HF-20	https://figshare.com/articles/dataset/EukProt_a_datab	C:178 [S:150, D:28], F:41, M:36, n:255
Haptista	Centroplasthelida								Choanocystis sp HF-7	https://figshare.com/articles/dataset/EukProt_a_datab	C:226 [S:126, D:100], F:10, M:19, n:255
Haptista	Centroplasthelida								Pterocystis sp 00344	https://figshare.com/articles/dataset/EukProt_a_datab	C:205 [S:176, D:29], F:16, M:34, n:255
Haptista	Centroplasthelida								Raphidiophrys heterophryoidea	https://figshare.com/articles/dataset/EukProt_a_datab	C:211 [S:170, D:41], F:19, M:25, n:255
Haptista	Haptophyta								Chrysochromulina tobinii	https://figshare.com/articles/dataset/EukProt_a_datab	C:137 [S:135, D:2], F:23, M:95, n:255
Haptista	Haptophyta								Diacronema sp CCMP2436	https://figshare.com/articles/dataset/EukProt_a_datab	C:195 [S:165, D:30], F:26, M:34, n:255
Haptista	Haptophyta								Emiliania huxleyi	https://figshare.com/articles/dataset/EukProt_a_datab	C:147 [S:106, D:41], F:40, M:68, n:255
Haptista	Haptophyta								Gephyrocapsa oceanica	https://figshare.com/articles/dataset/EukProt_a_datab	C:170 [S:143, D:27], F:36, M:49, n:255
Haptista	Haptophyta								Prymnesium parvum	https://figshare.com/articles/dataset/EukProt_a_datab	C:182 [S:153, D:29], F:18, M:55, n:255
Malawimonadida									Gefionella okellyi	https://figshare.com/articles/dataset/EukProt_a_datab	C:202 [S:158, D:44], F:5, M:48, n:255
Metamonada	Fornicata								Carpediemonas membranifera	https://figshare.com/articles/dataset/EukProt_a_datab	C:122 [S:96, D:26], F:31, M:102, n:255
Metamonada	Fornicata								Dysnectes brevis	https://figshare.com/articles/dataset/EukProt_a_datab	C:83 [S:83, D:0], F:32, M:140, n:255
Metamonada	Metamonada incertae sedis								Barthelona sp PAP020	https://figshare.com/articles/dataset/EukProt_a_datab	C:114 [S:93, D:21], F:19, M:122, n:255
Metamonada	Parabasalia								Trichomonas vaginalis	https://figshare.com/articles/dataset/EukProt_a_datab	C:136 [S:107, D:29], F:14, M:105, n:255
Metamonada	Parabasalia								Tritrichomonas foetus	https://figshare.com/articles/dataset/EukProt_a_datab	C:128 [S:102, D:26], F:13, M:114, n:255
Metamonada	Preaxostyla								Monocercomonoides exilis	https://figshare.com/articles/dataset/EukProt_a_datab	C:145 [S:124, D:21], F:18, M:92, n:255
Metamonada	Preaxostyla								Trimastix marina	https://figshare.com/articles/dataset/EukProt_a_datab	C:115 [S:111, D:4], F:13, M:127, n:255
TSAR	Sar	Alveolata	Apicomplexa						Plasmodium falciparum	https://figshare.com/articles/dataset/EukProt_a_datab	C:145 [S:142, D:3], F:8, M:102, n:255
TSAR	Sar	Alveolata	Apicomplexa						Toxoplasma gondii	https://figshare.com/articles/dataset/EukProt_a_datab	C:157 [S:157, D:0], F:21, M:77, n:255
TSAR	Sar	Alveolata	Ciliophora						Paramecium tetraurelia	https://figshare.com/articles/dataset/EukProt_a_datab	C:184 [S:82, D:102], F:9, M:62, n:255
TSAR	Sar	Alveolata	Ciliophora						Sterkiella histriomuscorum	https://figshare.com/articles/dataset/EukProt_a_datab	C:192 [S:150, D:42], F:9, M:54, n:255
TSAR	Sar	Alveolata	Ciliophora						Tetrahymena thermophila	https://figshare.com/articles/dataset/EukProt_a_datab	C:198 [S:187, D:11], F:8, M:49, n:255
TSAR	Sar	Alveolata	Colpodellida						Chromera velia	https://figshare.com/articles/dataset/EukProt_a_datab	C:169 [S:166, D:3], F:28, M:58, n:255
TSAR	Sar	Alveolata	Colpodellida						Vitrella brassicaformis	https://figshare.com/articles/dataset/EukProt_a_datab	C:200 [S:198, D:2], F:18, M:37, n:255
TSAR	Sar	Alveolata	Colponemida						Colponemidia sp Colp-10	https://figshare.com/articles/dataset/EukProt_a_datab	C:238 [S:227, D:11], F:7, M:10, n:255
TSAR	Sar	Alveolata	Colponemida						Colponemidia sp Colp-15	https://figshare.com/articles/dataset/EukProt_a_datab	C:228 [S:222, D:6], F:17, M:10, n:255
TSAR	Sar	Alveolata	Dinophyceae						Oxyrrhis marina	https://figshare.com/articles/dataset/EukProt_a_datab	C:164 [S:145, D:19], F:27, M:64, n:255
TSAR	Sar	Rhizaria							Bigelowiella natans	https://figshare.com/articles/dataset/EukProt_a_datab	C:186 [S:182, D:4], F:29, M:40, n:255
TSAR	Sar	Rhizaria							Brevimastigomonas motovehiculus	https://figshare.com/articles/dataset/EukProt_a_datab	C:140 [S:137, D:3], F:37, M:78, n:255
TSAR	Sar	Rhizaria							Lapot gusevi	https://figshare.com/articles/dataset/EukProt_a_datab	C:193 [S:112, D:81], F:31, M:31, n:255
TSAR	Sar	Rhizaria							Leptophrys vorax	https://figshare.com/articles/dataset/EukProt_a_datab	C:185 [S:118, D:67], F:37, M:33, n:255
TSAR	Sar	Rhizaria							Mataza sp D1	https://figshare.com/articles/dataset/EukProt_a_datab	C:199 [S:197, D:2], F:23, M:33, n:255
TSAR	Sar	Rhizaria							Nonionella stella	https://figshare.com/articles/dataset/EukProt_a_datab	C:245 [S:125, D:120], F:2, M:8, n:255
TSAR	Sar	Rhizaria							Paulinella chromatophora	https://figshare.com/articles/dataset/EukProt_a_datab	C:204 [S:186, D:18], F:29, M:22, n:255
TSAR	Sar	Rhizaria							Paulinella micropora	https://figshare.com/articles/dataset/EukProt_a_datab	C:173 [S:166, D:7], F:18, M:64, n:255
TSAR	Sar	Rhizaria							Plasmodiophora brassicae	https://figshare.com/articles/dataset/EukProt_a_datab	C:228 [S:227, D:1], F:8, M:19, n:255
TSAR	Sar	Stramenophiles							Cafeteria burkhardae	https://figshare.com/articles/dataset/EukProt_a_datab	C:172 [S:168, D:4], F:22, M:61, n:255
TSAR	Sar	Stramenophiles							Chattonella subsalsa	https://figshare.com/articles/dataset/EukProt_a_datab	C:184 [S:169, D:15], F:26, M:45, n:255
TSAR	Sar	Stramenophiles							Ectocarpus siliculosus	https://figshare.com/articles/dataset/EukProt_a_datab	C:217 [S:213, D:4], F:19, M:19, n:255
TSAR	Sar	Stramenophiles							Halocafeteria seosinensis	https://figshare.com/articles/dataset/EukProt_a_datab	C:214 [S:199, D:15], F:13, M:28, n:255
TSAR	Sar	Stramenophiles							Haloplacidia sp CaronLabIsolate	https://figshare.com/articles/dataset/EukProt_a_datab	C:164 [S:164, D:0], F:30, M:61, n:255
TSAR	Sar	Stramenophiles							Hondaea fermentalgiana	https://figshare.com/articles/dataset/EukProt_a_datab	C:225 [S:222, D:3], F:5, M:25, n:255
TSAR	Sar	Stramenophiles							MAST-04A sp MAST-4A-sp1	https://figshare.com/articles/dataset/EukProt_a_datab	C:188 [S:183, D:5], F:17, M:50, n:255
TSAR	Sar	Stramenophiles							Phaeodactylum tricornutum	https://figshare.com/articles/dataset/EukProt_a_datab	C:201 [S:197, D:4], F:15, M:39, n:255
TSAR	Sar	Stramenophiles							Phytopythium vexans	https://figshare.com/articles/dataset/EukProt_a_datab	C:229 [S:225, D:4], F:11, M:15, n:255
TSAR	Sar	Stramenophiles							Plasmopara halstedii	https://figshare.com/articles/dataset/EukProt_a_datab	C:230 [S:227, D:3], F:13, M:12, n:255
TSAR	Sar	Stramenophiles							Saprolegnia diclina	https://figshare.com/articles/dataset/EukProt_a_datab	C:239 [S:229, D:10], F:3, M:13, n:255
TSAR	Sar	Stramenophiles							Thalassiosira pseudonana	https://figshare.com/articles/dataset/EukProt_a_datab	C:188 [S:177, D:11], F:29, M:38, n:255

Supplementary Table 2. Details of placozoan iGluR protein sequences and proposed nomenclature.

T. adhaerens	Ensemble ID	Other IDs	Sebe Pedros	scRNA Data?	Used in Tree	Prevous name	Proposed nomenclature
	TriadG19383	evg1356554	Tadh_TriadG19383	N	evg1356554	AKDF1	AKDF1
	TriadG14565	evg1673038	Tadh_TriadG14565	Y	evg1673038	**AKDF2**	AKDF2
	TriadG25027	evg1036978	Tadh_TriadG25025	Y	evg1036978	**AKDF3**	AKDF3
	TriadG25027	evg1237846	Tadh_TriadG25027	Y	evg1237846	AKDF4	AKDF4
	TriadG3218	evg1324853	NA	N	evg1324853	GluE2	GluE1αA
	TriadG32461	evg9235	Tadh_TriadG32461	Y	evg9235	TGluE3	GluE1αB
	TriadG61396	XP_002117383.1	Tadh_TriadG61396	Y	XP_002117383.1	GluE4	GluE1αC
	TriadG55165	evg106810	Tadh_TriadG55165	Y	evg106810	GluE1	GluE1γ
	TriadG30612	XP 002116224.1	Tadh_TriadG30612	Y	XP_002116224.1	GluE6	GluE2α
	TriadP30609	evg4931	Tadh_TriadG30609	Y	evg4931	TGluE5	GluE2β
	TriadP18262	evg1416140	Tadh_TriadG18262	Y	evg1416140	TGluE10	GluE3α
	TriadG18488	N/A	Tadh_TriadG18488	N	TriadG18488	GluE8	GluE3βA
	TriadG18823	evg87016	Tadh_TriadG18823	Y	evg87016+TriadG18823	GluE7	GluE3βB
	TriadP18943	evg1037199	Tadh_TriadG18943	Y	evg1037199	GluE9	GluE3γ
	N/A	evg1807018	NA	N	evg1807018	N/A	GluE3δ

T. spH2	Ensemble ID	Other IDs	Sebe Pedros	scRNA Data?	Used in Tree	Prevous name	Proposed nomenclature
		TsH2_A0A369RQ89	TrH2_TrispH2_010896	Y	TsH2_A0A369RQ89	N/A	AKDF1
		TsH2_A0A369S2U4	TrH2_TrispH2_007748	Y	TsH2_A0A369S2U4	N/A	AKDF2
		TsH2_A0A369RNV1	TrH2_TrispH2_011511	Y	TsH2_A0A369RNV1	N/A	AKDF3
		TsH2_A0A369S4U1	TrH2_TrispH2_007722	Y	TsH2_A0A369S4U1	N/A	AKDF4
		TsH2_A0A369RV60	TrH2_TrispH2_009956	Y	TsH2_A0A369RV60	N/A	GluE1αB
		TsH2_A0A369RSZ7	TrH2_TrispH2_010734	Y	TsH2_A0A369RSZ7	N/A	GluE1γ
		TsH2_A0A369SKY4	TrH2_TrispH2_000862	Y	TsH2_A0A369SKY4	N/A	GluE2α
		TsH2_A0A369SKW2	TrH2_TrispH2_000861	Y	TsH2_A0A369SKW2	N/A	GluE2β
		TsH2_A0A369SJG5	TrH2_TrispH2_001055	Y	TsH2_A0A369SJG5	N/A	GluE3α
		TsH2_A0A369SI83	TrH2_TrispH2_001054	Y	TsH2_A0A369SI83	N/A	GluE3β
		TsH2_A0A369SIE5	TrH2_TrispH2_001056	Y	TsH2_A0A369SIE5	N/A	GluE3γ
		TsH2_A0A369RZR6	TrH2_TrispH2_007119	Y	TsH2_A0A369RZR6	N/A	GluE3δ

H. hongkongensis	Ensemble ID	Other IDs	Sebe Pedros	scRNA Data?	Used in Tree	Prevous name	Proposed nomenclature
		TR3071_c1_g1_i8_m.6854	Hhon_g01988.t1	Y	TR3071_c1_g1_i8_m.6854	N/A	AKDF1
		TR15883_c3_g1_i1_m.30362	Hhon_g02946.t1	Y	TR15883_c3_g1_i1_m.30362	N/A	AKDF2
		TR21743_c0_g2_i1_m.42230	Hhon_g02972.t1	Y	TR21743_c0_g2_i1_m.42230	N/A	AKDF3
		TR21198_c2_g1_i8_m.40896	Hhon_g02971.t1	Y	TR21198_c2_g1_i8_m.40896	N/A	AKDF4
		TR13015_c0_g2_i3_m.25087	NA	N	TR13015_c0_g2_i3_m.25087	N/A	GluE1αC
		TR30216_c5_g1_i2_m.64944	NA	N	TR30216_c5_g1_i2_m.64944	N/A	GluE1αE
		TR9210_c0_g1_i1_m.20124	NA	N	TR9210_c0_g1_i1_m.20124	N/A	GluE1β
		TR30549_c0_g1_i1_m.66150	NA	N	TR30549_c0_g1_i1_m.66150	N/A	GluE3α
		TR15638_c1_g1_i3_m.29640	NA	N	TR15638_c1_g1_i3_m.29640	N/A	GluE3β
		TR19588_c2_g2_i3_m.37163	Hhon_g04079.t1	Y	TR19588_c2_g2_i3_m.37163	N/A	GluE3γ

C. collaboinventa	Ensemble ID	Other IDs	Sebe Pedros	scRNA Data?	Used in Tree	Prevous name	Proposed nomenclature
		HoiH23_PlH23_003663	HoiH23_PlH23_003663	Y	HoiH23_PlH23_003663	N/A	AKDF1
		HoiH23_PlH23_010905	HoiH23_PlH23_010905	Y	HoiH23_PlH23_010905	N/A	AKDF2
		HoiH23_PlH23_011441	HoiH23_PlH23_011441	Y	HoiH23_PlH23_011441	N/A	AKDF3
		HoiH23_PlH23_007108	HoiH23_PlH23_007108	Y	HoiH23_PlH23_007108	N/A	AKDF4
		HoiH23_PlH23_006517	HoiH23_PlH23_006517	Y	HoiH23_PlH23_006517	N/A	GluE1αC
		HoiH23_PlH23_006518	HoiH23_PlH23_006518	Y	HoiH23_PlH23_006518	N/A	GluE1αD
		HoiH23_PlH23_006519	HoiH23_PlH23_006519	Y	HoiH23_PlH23_006519	N/A	GluE1αE
		HoiH23_PlH23_006516	HoiH23_PlH23_006516	Y	HoiH23_PlH23_006516	N/A	GluE1β
		HoiH23_PlH23_005700	HoiH23_PlH23_005700	Y	HoiH23_PlH23_005700	N/A	GluE2α
		HoiH23_PlH23_000801	HoiH23_PlH23_000801	Y	HoiH23_PlH23_000801	N/A	GluE3α
		HoiH23_PlH23_011585	HoiH23_PlH23_011585	Y	HoiH23_PlH23_011585	N/A	GluE3β
		HoiH23_PlH23_000803	HoiH23_PlH23_000803	Y	HoiH23_PlH23_000803	N/A	GluE3γ

Supplementary Table 3. Docking energies for receptor-ligand interactions.

Table S3a to c. MC energy minimization data for GluE1aA-amino acid ligand interactions in kcal/mol (residues numbered according to mature rat GluA2)

a. Receptor interactions with ligand

	wt GLY	m3 GLY	wt GLU	m3 GLU
Ligand-receptor H-bonds	-2.66	-1.69	-2.06	-2.33
Ligand-receptor van der Waals	-10.90	-9.35	-19.12	-16.77
Ligand-receptor electrostatic	-11.01	-7.31	-7.61	-8.51
Ligand-receptor desolvation	18.65	15.84	30.53	24.60
Ligand-receptor total	-5.92	-2.51	1.74	-3.01

b. Energy contributions from specific receptor residues (GLY)

Top attractive residues				Top repulsive residues			
Res.	Total	Bckbn	Sdchn	Res.	Total	Bckbn	Sdchn
**Wildtype**							
R485	-5.67	-0.11	-5.56	S654	0.51	-0.03	0.54
D705	-2.78	0.11	-2.89	L479	0.36	0.18	0.17
K728	-0.27	0.00	-0.27	E484	0.36	0.00	0.36
Y450	-0.26	-0.03	-0.23	L650	0.33	0.21	0.12
Y657	-0.20	-0.18	-0.02	G704	0.33	0.29	0.04
S480	-0.17	-0.05	-0.13	G451	0.30	0.43	-0.13
R754	-0.16	0.00	-0.16	D447	0.30	0.00	0.30
D402	-0.15	0.00	-0.15	E656	0.25	-0.10	0.35
K671	-0.14	0.00	-0.14	Q498	0.24	0.00	0.24
K738	-0.13	0.00	-0.13	F732	0.23	-0.21	0.45
**m3 variant**							
R485	-4.25	-0.04	-4.21	Y732	0.60	0.05	0.55
D705	-2.33	-0.03	-2.29	K728	0.54	0.00	0.54
D730	-0.39	-0.07	-0.32	P478	0.53	0.43	0.10
Y450	-0.14	-0.05	-0.09	E656	0.38	0.08	0.30
K738	-0.13	0.00	-0.13	L479	0.38	0.21	0.17
R684	-0.12	0.00	-0.12	S654	0.35	0.44	-0.09
R443	-0.12	0.00	-0.12	E484	0.34	0.00	0.34
R692	-0.12	0.00	-0.12	S480	0.31	0.36	-0.05
G462	-0.11	-0.07	-0.04	D447	0.29	0.00	0.29
K710	-0.11	0.00	-0.11	G704	0.28	0.22	0.06

c. Energy contributions from specific receptor residues (GLU)

Top attractive residues				Top repulsive residues			
Res.	Total	Bckbn	Sdchn	Res.	Total	Bckbn	Sdchn
**Wildtype**							
R485	-7.24	-0.10	-7.15	D705	2.08	0.34	1.74
K728	-0.76	0.00	-0.76	S480	1.00	0.52	0.48
Y450	-0.51	0.06	-0.57	E656	0.97	0.08	0.89
Y657	-0.40	-0.38	-0.02	N686	0.81	-0.05	0.87
R692	-0.31	0.00	-0.31	D730	0.74	0.03	0.71
G462	-0.27	-0.18	-0.08	S654	0.70	-0.44	1.14
V649	-0.26	-0.24	-0.02	L479	0.60	0.11	0.49
K671	-0.24	0.00	-0.24	D402	0.58	-0.08	0.66
R684	-0.21	0.02	-0.23	E484	0.44	0.00	0.44
G704	-0.20	0.21	-0.41	D447	0.41	0.00	0.41
**m3 variant**							
R485	-6.43	-0.05	-6.39	D705	1.40	0.05	1.36
K728	-0.98	0.00	-0.98	D730	0.93	0.00	0.93
G653	-0.87	-0.04	-0.83	S480	0.63	0.42	0.21
T655	-0.51	-0.20	-0.31	L479	0.52	0.21	0.31
G462	-0.35	-0.29	-0.06	E656	0.49	-0.03	0.52
R692	-0.24	0.00	-0.24	E484	0.40	0.00	0.40
A403	-0.23	-0.23	0.00	G451	0.39	0.52	-0.13
Q498	-0.21	0.00	-0.21	N686	0.36	0.03	0.33
K671	-0.19	0.00	-0.19	D447	0.32	0.00	0.32
V463	-0.18	-0.17	-0.01	T461	0.27	0.24	0.02

Table S3d and e. MC energy minimization data in kcal/mol for GluE1aA-spermine interactions (residues numbered according to the full length GluE1aA sequence)

d. Receptor interactions with ligand (step position 32 for wildtype and 34 for S642N)

	wt	S642N
Ligand-receptor H-bonds	-2.57	-2.27
Ligand-receptor van der Waals	-18.06	-15.66
Ligand-receptor electrostatic	-23.34	-30.32
Ligand-receptor desolvation	27.59	27.93
Ligand-receptor total	-16.38	-20.32

e. Energy contributions from specific receptor residues (Spermine)

Top attractive residues					Top repulsive residues				
Sub.	Res.	Total	Bckbn	Sdchn	Sub.	Res.	Total	Bckbn	Sdchn
**Wildtype**									
4	D646	-6.70	-0.63	-6.08	3	R647	1.86	0.32	1.54
3	D646	-3.59	-1.73	-1.86	4	R647	1.46	0.00	1.46
1	D646	-2.63	-0.46	-2.17	3	T648	0.67	0.16	0.51
4	Q643	-1.73	-1.58	-0.16	2	R647	0.59	0.01	0.58
1	Q643	-1.44	-1.60	0.16	3	G644	0.56	-0.24	0.80
2	D646	-1.43	-0.36	-1.07	1	R647	0.51	-0.16	0.67
1	S642	-0.78	-1.21	0.43	3	R650	0.49	0.00	0.49
3	A645	-0.72	-0.81	0.09	1	R650	0.48	0.00	0.48
3	D587	-0.55	0.00	-0.55	3	S642	0.42	-0.14	0.56
4	E622	-0.52	0.00	-0.52	3	K591	0.37	0.00	0.37
**S642N variant**									
1	D646	-2.97	-0.72	-2.25	1	R647	1.35	0.17	1.18
2	D646	-2.58	-0.51	-2.07	2	R647	1.06	0.09	0.97
4	D646	-2.57	-0.67	-1.90	1	R650	0.91	0.00	0.91
1	N642	-2.20	-1.66	-0.54	3	R647	0.61	-0.21	0.82
3	D646	-2.09	-0.53	-1.56	4	R647	0.37	-0.36	0.73
3	N642	-2.07	-0.63	-1.44	4	R650	0.34	0.00	0.34
4	Q643	-1.31	-1.18	-0.13	2	K591	0.33	0.00	0.33
1	Q643	-1.20	-1.18	-0.02	2	R650	0.33	0.00	0.33
2	N642	-1.05	-0.90	-0.15	3	R650	0.32	0.00	0.32
4	N642	-1.00	-0.38	-0.62	1	K591	0.31	0.00	0.31

Supplementary Table 4. Statistical parameters for analyzed data.

Table S4a. Average permeability ratios for variants of the T. adhaerens GluE1αA receptor under monovalent bi-ionic conditions.

		WT	SQ	SN	SR	DA	SQDA
	**pNa/pNa**	1.00±0.02^{a}	1.00±0.07^{a}	1.00±0.08^{a}	1.00±0.02^{a}	1.00±0.04^{a}	1.00±0.10^{a}
	**pLi/pNa**	0.80±0.02^{b}	1.12±0.15^{a,***}	0.85±0.04^{b}	1.02±0.03^{a,***}	0.98±0.03^{a,***}	0.98±0.10^{a,**}
	**pK/pNa**	1.06±0.02^{c}	1.00±0.11^{a}	0.99±0.10^{ab}	0.91±0.07^{b,**}	1.10±0.04^{b,*}	1.14±0.09^{b,*}
	**pCs/pNa**	0.94±0.02^{d}	1.05±0.09^{a,*}	1.02±0.09^{a,*}	0.83±0.04^{c,***}	0.99±0.04^{a,*}	1.13±0.08^{b,**}
ANOVAs	F-statistic	204.87	1.48	4.32	19.94	20.81	6.68
	p value	1.0E-14	2.5E-01	1.2E-02	1.4E-06	4.4E-07	1.3E-03

a,b,c,d: $p<0.05$ for Tukey post hoc tests for single variants within bi-ionic conditions

***:** $p<0.05$ for 2-sample T-test for each variant relative to wildtype under the same bi-ionic conditions

****:** $p<0.005$ for 2-sample T-test for each variant relative to wildtype under the same bi-ionic conditions

*****:** $p<0.0005$ for 2-sample T-test for each variant relative to wildtype under the same bi-ionic conditions

Table S4b. Average permeability ratios for various variants of the T. adhaerens GluE1αA receptor under monovalent-divalent cationic conditions.

		WT	SQ	SN	SR	DA	SQDA
	**pCa/pNa**	0.81±0.14^{ab}	9.67±0.67^{a,***}	3.37±0.62^{ab,***}	1.62±0.32^{a,***}	0.61±0.07^{a,**}	9.50±0.41^{a,***}
	**pCa/pLi**	1.01±0.28^{a}	10.79±0.54^{b,***}	3.38±0.23^{ab,***}	1.90±0.27ab'***	0.59±0.05^{a,**}	7.08±0.74^{b,***}
	**pCa/pK**	0.67±0.11^{b}	6.58±0.60^{c,***}	3.06±0.19^{a,***}	2.26±0.31^{bc,***}	0.61±0.04^{a,**}	6.33±0.47^{b,***}
	**pCa/pCs**	1.14±0.10^{a}	7.85±0.56^{d,***}	4.11±0.81^{b,**}	2.53±0.39^{c,**}	0.99±0.06^{b,**}	6.46±1.14^{b,***}
ANOVAs	F-statistic	10.19	60.14	4.09	9.13	63.96	23.3
	p value	9.5E-05	1.4E-11	1.8E-02	3.3E-04	1.2E-12	2.7E-07

a,b,c: $p<0.05$ for Tukey post hoc tests for single variants within bi-ionic conditions

****:** $p<0.005$ for 2-sample T-test for each variant relative to wildtype under the same bi-ionic conditions

*****:** $p<0.0005$ for 2-sample T-test for each variant relative to wildtype under the same bi-ionic conditions

Supplementary Table 5. Primers used for cloning and mutagenesis of iGluRs, and accession numbers of cloned *T. adhaerens* receptors.

Cloning primers				
Name	Sequence (5'-3')	Restriction Enzymes	cDNA PCR succesful?	NCBI Acc. No.
TadGluE1αA_F1	CAGCTGATACGTGAATTTCGTGGC			
TadGluE1αA_R1	GGCACAGAATACTGATGCATTTGTAGC			
TadGluE1αA_F2	TATATAAGATCTGCCGCCACCATGGTAAAATCGAATTTCATATACTATT	BglII		
TadGluE1αA_R2	TATATAGTCGACTTATAAGTACGATATCCGTATCATCTC	SalI	Yes	PP886186
TadGluE1αB_F1	CCGGTCCATCAACTATCCTATCCATTG			
TadGluE1αB_R1	CAGCTTTCTAACAGTTAGACCATTC			
TadGluE1αB_F2	TATATAGTCGACGCCGCCACCATGAATCATATCATTGATTCAGGTTCG	SalI		
TadGluE1αB_R2	TATATAGGATCCTTATATTGCGGTCGGTACTGG	BamHI	Yes	PP886187
TadGluE1γ_F1	CGCTGCCATTGACTGGTAGTAAG			
TadGluE1γ_R1	GTTCATAATCCTACCGATGTAACAGC			
TadGluE1γ_F2	TATATAGAATTCGCCGCCACCATGAAATCGCTTGTGATAGCAAAT	EcoRI		
TadGluE1γ_R2	TATATAGGATCCCTACACCATGACTCCAATTTTATTAGAG	BamHI	Yes	PP886188
TadGluE2β_F1	CACTTCTTGGCAGCCGCAATACAC			
TadGluE2β_R1	CCTGATTCCTTAACAATGTTGTGTCTAG			
TadGluE2β_F2	TATATAGAATTCGCCGCCACCATGGCTAGGCAAAGATTAATATACCTCG	EcoRI		
TadGluE2β_R2	TATATAGGATCCTCAGACTTCCACTGCCGTTTGC	BamHI	Yes	PP886189
TadGluE3α_F1	GAATTCTAGATAGTGCAAACACGACTTG			
TadGluE3α_R1	CAAGGCTTCGACGGGAATCGGTG			
TadGluE3α_F2	TATATAGTCGACGCCGCCACCATGATGAAACCCTTAAATTCAAGTTTTC	SalI		
TadGluE3α_R2	TATATAGGATCCTTAGCACTGTATTTGAGAAGAAGCTC	BamHI	Yes	PP886190
TadGluE3βA_F1	ACAATATCACTGCTCAATCCGG			
TadGluE3βA_R1	CTATTATCGGCGTACTGCAAGTATATGG			
TadGluE3βA_F2	TATATAAGATCTGCCGCCACCATGCGCGATAGAATCTGGGCTATTATAG	BglII		
TadGluE3βA_R2	TATATAGTCGACTTAACCGATAAGTTTAATATCACTATTTTTTTC	SalI	No	NA
TadGluE3γ_F1	CGATATATTTCTTGGCCTCGTAACAAGG			
TadGluE3γ_R1	GCCAGGAATTTGCAATATGATTTTACTCC			
TadGluE3γ_F2	TATATAAGATCTGCCGCCACCATGGTCAATACTCTAGTCCACCG	BglII		
TadGluE3γ_R2	TATATAGTCGACTCAACATTCAATTGGAGATGCTAGC	SalI	Yes	PP886191
TadGluE3δ_F1	CCTGCCGTCCAATTTCGGTCAAG			
TadGluE3δ_R1	CAAGATCGTTTTGCTGATTACAGTAGTC			
TadGluE3δ_F2	TATATAGTCGACGCCGCCACCATGATGAATATTATTTCCTTGATTAAAT	SalI		
TadGluE3δ_R2	TATATACCCGGGTTAAACTGGAGACGGTCGGATGATG	TspMI	No	NA
TadAKDF1_F1	GTGGATAGAATAGACATCGACATCACC			
TadAKDF1_R1	GATTGGTTTTTCCTCTGCCCTTGG			
TadAKDF1_F2	TATATAGTCGACGCCGCCACCATGCCAATATCCAAATTGGTTATC	SalI		
TadAKDF1_R2	TATATAGGATCCTTATTCAAATAAAGCATTACCGTGG	BamHI	Yes	PP886192
TadAKDF2_F1	GCGATCTAGCTACGTGAACATTCGTAAC			
TadAKDF2_R1	CCGCTTGCAAACTCTTAAATATGGTTAG			
TadAKDF2_F2	TATATAAGATCTGCCGCCACCATGAGGCCGATAAGGTTCTCTATTC	BglII		
TadAKDF2_R2	TATATAGTCGACCTAATCGCCGGCATCGTTGCCAAATC	SalI	Yes	PP886193
TadAKDF3_F1	CAACGTCAACGAACCTCTTCGTGGCTAC			
TadAKDF3_R1	GTCTTACATCTGTTGATCTAGG			
TadAKDF3_F2	TATATAGAGCTCGCCGCCACCATGAAGTACTTATACAGCAAATATTGG	SacI		
TadAKDF3_R2	TATATAGGATCCTCAAATTTGTGCTTTATG	BamHI	Yes	PP886194
TadAKDF4_F1	GCATTACATATCAGGGAAATCAGATAAG			
TadAKDF4_R1	CGGTAAATCATGATCATCCGTTCCC			
TadAKDF4_F2	TATATAAGATCTGCCGCCACCATGAAAAAAATGAAAATTATAGGACTG	BglII		
TadAKDF4_R2	TATATAGTCGACTTATTCCTTTAAATCATCAACTCG	SalI	Yes	PP886195

Mutagenesis Primers	
HumGRIA2_Q607S-F	TCTCTCGGCGCCTTCATGTCCCAGGGATGTGATATTTCT
HumGRIA2_Q607S-R	AGAAATATCACATCCCTGGGACATGAAGGCGCCGAGAGA
TadGluE1αA_S642N-F	ACATTGGGATCCTTTCTGAACCAAGGGGCTGATAGAACA
TadGluE1αA_S642N-R	TGTTCTATCAGCCCCTTGGTTCAGAAAGGATCCCAATGT
TadGluE1αA_S642R-F	ACATTGGGATCCTTTCTGAGACAAGGGGCTGATAGAACA
TadGluE1αA_S642R-R	TGTTCTATCAGCCCCTTGTCTCAGAAAGGATCCCAATGT
TadGluE1αA_D646A-F	TTTCTGTCCCAAGGGGCTGCCAGAACACCAAGATCGATA
TadGluE1αA_D646A-R	TATCGATCTTGGTGTTCTGGCAGCCCCTTGGGACAGAAA
TadGluE1αA_SQ_DA-F	TTTCTGCAACAAGGGGCTGCCAGAACACCAAGATCGATA
TadGluE1αA_SQ_DA-R	TATCGATCTTGGTGTTCTGGCAGCCCCTTGTTGCAGAAA
TadGluE1αA_SI-GT_F	GATATGGAGTTTTAGCGAATGGCAGCACCGAATATTTCTTCTTGAACAC
TadGluE1αA_SI-GT_R	GTGTTCAAGAAGAAATATTCGGTGCTGCCATTCGCTAAAACTCCATATC
TadGluE1αA_F-Y_F	GTTTTAAAGAAGACAGCTATGGCTTGGCGCTACCAAA
TadGluE1αA_F-Y_R	TTTGGTAGCGCCAAGCCATAGCTGTCTTCTTTAAAAC

References

1. Morel, B., et al., *GeneRax: a tool for species-tree-aware maximum likelihood-based gene family tree inference under gene duplication, transfer, and loss*. Molecular biology and evolution, 2020. **37**(9): p. 2763-2774.
2. Minh, B.Q., et al., *IQ-TREE 2: new models and efficient methods for phylogenetic inference in the genomic era*. Molecular biology and evolution, 2020. **37**(5): p. 1530-1534.
