# Supplementary material for: Evolution of iGluR ligand specificity, polyamine regulation, and ion selectivity inferred from a placozoan epsilon receptor
Source: Commun Biol. 2025 Jul 3;8:994. doi: 10.1038/s42003-025-08402-3 (PMC12226733; doi:10.1038/s42003-025-08402-3)
Supplement: Supplementary file 3 — Description of Additional Supplementary Files [file 42003_2025_8402_MOESM3_ESM.pdf]

Description of Additional Supplementary Files

File name: Supplementary Data 1

Description: Protein alignment of TadGluE1aA and rat AMPA2 sequences.

File name: Supplementary Data 2

Description: CLANS output file for Supplementary Figure 4.

File name: Supplementary Data 3

Description: Raw and averaged data used to generate all plots in the manuscript.
