# Supplementary material for: Evolution of iGluR ligand specificity, polyamine regulation, and ion selectivity inferred from a placozoan epsilon receptor
Source: Commun Biol. 2025 Jul 3;8:994. doi: 10.1038/s42003-025-08402-3 (PMC12226733; doi:10.1038/s42003-025-08402-3)
Supplement: Supplementary file 4 — Supplementary Data 1 [file 42003_2025_8402_MOESM4_ESM.docx]

**Supplementary File 1: Global alignment of T. adhaerens GluE1aA and mature rat GluA2 (UniProt ID number P19491)**

Residues listed in Table S3 are back-colored light purple.

GE1aA 1 MVKSNFIYYLLLFQAAGLIFAVTANVETTYRLGILLPRGYTKIERAIQLAIDMINENEIA 60

V SN I ++G L PRG + A ++ + + +E

rGA2 1 -VSSNSI-----------------------QIGGLFPRGADQEYSAFRVGMVQFSTSEF- 35

GE1aA 61 DIKLNHSRLSTTFHTADLYSPYDNFHKACNAMKEEIVAIIGPLVSGATIGAQFACSTLNM 120

RL+ ++ + + + C+ + AI G + C TL++

rGA2 36 -------RLTPHIDNLEVANSFAVTNAFCSQFSRGVYAIFGFYDKKSVNTITSFCGTLHV 88

GE1aA 121 PHIAP-FATDANLANNPSYTYLLRMLPVSTIESLAIASFIEYYGWTKVAILASNTDFGVS 179

I P F TD ++ ++++M P ++ A+ S IEYY W K A L ++D G+S

rGA2 89 SFITPSFPTDG------THPFVIQMRP--DLKG-ALLSLIEYYQWDKFAYLY-DSDRGLS 138

GE1aA 180 VLSKFREIASRKSWRILAFELFKIDQAGNLLDIETNLQSIKKSGARIVIINCLTSEALKI 239

L + A+ K W++ A + I+ + Q ++ R VI++C + I

rGA2 139 TLQAVLDSAAEKKWQVTAINVGNINNDKKDETYRSLFQDLELKKERRVILDCERDKVNDI 198

GE1aA 240 FERAREMGMMDSGWCWIAVDAIAAETSQLP-----PNLNGLIGLAYHNNR-GKLYQNVSA 293

++ +G G+ +I + + L N++G + Y ++ K + S

rGA2 199 VDQVITIGKHVKGYHYIIANLGFTDGDLLKIQFGGANVSGFQIVDYDDSLVSKFIERWST 258

GE1aA 294 ----RYYLRYNETIEPLYLHYFDSVLAVAYGIQAMVREG-KLPKAAKVICDKSNPK-PWK 347

Y + TI+ +D+V + + + ++ ++ + +NP PW

rGA2 259 LEEKEYPGAHTATIKYTSALTYDAVQVMTEAFRNLRKQRIEISRRGNAGDCLANPAVPWG 318

GE1aA 348 QGHQMFHFIKKASGPSTTSSINFNANGGPSNVGYDIINLHGQSWAKVGSWTNHRNLILDR 407

QG ++ +K+ + +I F+ NG N +I+ L K+G W+ +++

rGA2 319 QGVEIERALKQVQVEGLSGNIKFDQNGKRINYTINIMELKTNGPRKIGYWSEVDKMVVTL 378

GE1aA 408 NRIYFLNGVNHILDSGSFIGG---KTLTVTTILDAPFTMADRDYAIT--GRKYRGYIIDL 462

L SG+ G KT+ VTTIL++P+ M +++ + +Y GY +DL

rGA2 379 TE----------LPSGNDTSGLENKTVVVTTILESPYVMMKKNHEMLEGNERYEGYCVDL 428

GE1aA 463 LDEMSKNLNFTYKIRIVADGQYGSQYTDKNGALKWTGVIGEVIDGIADMAAAPLSITPER 522

E++K+ F YK+ IV DG+YG++ D W G++GE++ G AD+A APL+IT R

rGA2 429 AAEIAKHCGFKYKLTIVGDGKYGARDADTK---IWNGMVGELVYGKADIAIAPLTITLVR 485

GE1aA 523 QQALDFTMPFMNQGLTVLTLTKKNEANSLFQAFLPLKIEVWIGILISLIVVAIATTCMNR 582

++ +DF+ PFM+ G++++ + +F PL E+W+ I+ + I V++ ++R

rGA2 486 EEVIDFSKPFMSLGISIMIKKPQKSKPGVFSFLDPLAYEIWMCIVFAYIGVSVVLFLVSR 545

GE1aA 583 WSPFDYYGKAAEKLQMLEDRYQSESKLQHNYDYWQMEKEEALEAFSFGNTLWYTLGSFLS 642

+SP++++ + ED +++S E+ F N+LW++LG+F+

rGA2 546 FSPYEWH------TEEFEDGRETQSS-------------ESTNEFGIFNSLWFSLGAFMQ 586

GE1aA 643 QGADRTPRSISARLITAIWWLSSVIIIATYTANLTAFLTVSSLQTTFNSLHDLAKSSGMG 702

QG D +PRS+S R++ +WW ++III++YTANL AFLTV + + S DL+K + +

rGA2 587 QGCDISPRSLSGRIVGGVWWFFTLIIISSYTANLAAFLTVERMVSPIESAEDLSKQTEIA 646

GE1aA 703 YGVLANSSIEYFFLNTAVSPYQEMKHNLKN------VRSNMEGVQRVLSSTTEHYAYIGD 756

YG L + S + FF + ++ + +M +++ VR+ EGV RV S + YAY+ +

rGA2 647 YGTLDSGSTKEFFRRSKIAVFDKMWTYMRSAEPSVFVRTTAEGVARVRKSKGK-YAYLLE 705

GE1aA 757 AAVLKYAKSQY-CNLTTVG-SFKEDSFGLALPKESLYWKEVSIQILRFREEGFLETLQTK 814

+ + +Y + + C+ VG + +G+A PK S V++ +L+ E+G L+ L+ K

rGA2 706 STMNEYIEQRKPCDTMKVGGNLDSKGYGIATPKGSSLGNAVNLAVLKLNEQGLLDKLKNK 765

GE1aA 815 WF--EGKCKA----VSESTSHDPVKFESLIGVFYILLATVGFSFAILITEWIVAAIKDTR 868

W+ +G+C + E TS + ++ GVFYIL+ +G + + + E+ + + +

rGA2 766 WWYDKGECGSGGGDSKEKTS--ALSLSNVAGVFYILVGGLGLAMLVALIEFCYKSRAEAK 823

GE1aA 869 RHYMKISFAGAIKKRFLYIINDSFSATFCKDKTDRCNTGELSVTKTKRTSTLI 921

R MK++ + IN S S T + + K I

rGA2 824 R--MKVA-------KNPQNINPSSSQNSQNFATYKEGYNVYGIESVK-----I 862
