# Supplementary material for: Evolution of iGluR ligand specificity, polyamine regulation, and ion selectivity inferred from a placozoan epsilon receptor
Source: Commun Biol. 2025 Jul 3;8:994. doi: 10.1038/s42003-025-08402-3 (PMC12226733; doi:10.1038/s42003-025-08402-3)
Supplement: Supplementary file 7 — Reporting Summary [file 42003_2025_8402_MOESM7_ESM.pdf]

nature portfolio

Corresponding author(s): Adriano Senatore

Last updated by author(s): Jun 6, 2025

# Reporting Summary

Nature Portfolio wishes to improve the reproducibility of the work that we publish. This form provides structure for consistency and transparency in reporting. For further information on Nature Portfolio policies, see our Editorial Policies and the Editorial Policy Checklist.

## Statistics

For all statistical analyses, confirm that the following items are present in the figure legend, table legend, main text, or Methods section.

| n/a | Confirmed | |
|---|---|---|
| ☐ | ☒ | The exact sample size (*n*) for each experimental group/condition, given as a discrete number and unit of measurement |
| ☐ | ☒ | A statement on whether measurements were taken from distinct samples or whether the same sample was measured repeatedly |
| ☐ | ☒ | The statistical test(s) used AND whether they are one- or two-sided<br>*Only common tests should be described solely by name; describe more complex techniques in the Methods section.* |
| ☒ | ☐ | A description of all covariates tested |
| ☐ | ☒ | A description of any assumptions or corrections, such as tests of normality and adjustment for multiple comparisons |
| ☐ | ☒ | A full description of the statistical parameters including central tendency (e.g. means) or other basic estimates (e.g. regression coefficient) AND variation (e.g. standard deviation) or associated estimates of uncertainty (e.g. confidence intervals) |
| ☐ | ☒ | For null hypothesis testing, the test statistic (e.g. *F*, *t*, *r*) with confidence intervals, effect sizes, degrees of freedom and *P* value noted<br>*Give P values as exact values whenever suitable.* |
| ☒ | ☐ | For Bayesian analysis, information on the choice of priors and Markov chain Monte Carlo settings |
| ☒ | ☐ | For hierarchical and complex designs, identification of the appropriate level for tests and full reporting of outcomes |
| ☒ | ☐ | Estimates of effect sizes (e.g. Cohen's *d*, Pearson's *r*), indicating how they were calculated |

*Our web collection on statistics for biologists contains articles on many of the points above.*

## Software and code

Policy information about availability of computer code

| | |
|---|---|
| Data collection | The whole-cell patch clamp electrophysiology data was collected using pCLamp 11 Software. Structural modeling was done using Alphafold versions 2 and 3, and docking experiments were done with the program ZMM. Phylogenetic analyses were done using the programs BUSCO, HMMER, BLAST, SmartBLAST, InterPro, TMHMM, CD-HIT, CLANS, GeneRax, MAFFT, trimAl, FASconCAT, and IQ-TREE.<br>structures were analyzed and visualized using ChimeraX |
| Data analysis | pClamp 11 Software was used to analyze the electrophysiological recordings of the ion channels, along with Origin version 7 and Microsoft Excel. Molecular structures were analyzed (and visualized) using the UCSD ChimeraX and PyMOL. All figures were created using Adobe Illustrator. |

For manuscripts utilizing custom algorithms or software that are central to the research but not yet described in published literature, software must be made available to editors and reviewers. We strongly encourage code deposition in a community repository (e.g. GitHub). See the Nature Portfolio guidelines for submitting code & software for further information.

## Data

Policy information about availability of data

All manuscripts must include a data availability statement. This statement should provide the following information, where applicable:

- Accession codes, unique identifiers, or web links for publicly available datasets
- A description of any restrictions on data availability
- For clinical datasets or third party data, please ensure that the statement adheres to our policy

All data is available in the main text, supplementary figures, Supplementary Tabes S1 to S6, Supplementary Files 1 and 2, and in a Zenodo repository that is available though this link: https://zenodo.org/doi/10.5281/zenodo.11521256

## Human research participants

Policy information about studies involving human research participants and Sex and Gender in Research.

| | |
|---|---|
| Reporting on sex and gender | N/A |
| Population characteristics | N/A |
| Recruitment | N/A |
| Ethics oversight | N/A |

Note that full information on the approval of the study protocol must also be provided in the manuscript.

# Field-specific reporting

Please select the one below that is the best fit for your research. If you are not sure, read the appropriate sections before making your selection.

☒ Life sciences ☐ Behavioural & social sciences ☐ Ecological, evolutionary & environmental sciences

For a reference copy of the document with all sections, see nature.com/documents/nr-reporting-summary-flat.pdf

# Life sciences study design

All studies must disclose on these points even when the disclosure is negative.

| | |
|---|---|
| Sample size | Electrophysiology:<br>No statistical methods were used to determine the sample size. In all experiments we sought to exceed the minimum required sample sizes based on our own experience recording the ion channels and their reproducible biological responses. |
| Data exclusions | Electrophysiology data:<br>Data was excluded if the recorded traces showed an obvious defect, for example caused by losing adhesion of the recording electrode with the cell membrane during a patch recording, or if the perfusion system became clogged and failed to deliver appropriate solutions. Recorded cells that died or showed strong decay in current amplitudes were also excluded. |
| Replication | Electrophysiology data:<br>Each of the in vitro expressed ion channels and mutants were recorded over multiple days (i.e., independent transfections). The recording solutions used in the perfusion system were always applied in the same order, but their placement in the different lines of the perfusion system was changed to rule out potential differences in flow rate. |
| Randomization | Electrophysiology experiments:<br>HEK-293T cells were all transfected using consistent protocols, and cells expressing the reporter fluorescent protein were randomly selected for patch clamp recording. |
| Blinding | No blinding was performed on any of the mentioned experiments. |

# Reporting for specific materials, systems and methods

We require information from authors about some types of materials, experimental systems and methods used in many studies. Here, indicate whether each material, system or method listed is relevant to your study. If you are not sure if a list item applies to your research, read the appropriate section before selecting a response.

## Materials & experimental systems

| n/a | Involved in the study |
| --- | --- |
| ☒ | ☐ Antibodies |
| ☐ | ☒ Eukaryotic cell lines |
| ☒ | ☐ Palaeontology and archaeology |
| ☒ | ☐ Animals and other organisms |
| ☒ | ☐ Clinical data |
| ☒ | ☐ Dual use research of concern |

## Methods

| n/a | Involved in the study |
| --- | --- |
| ☒ | ☐ ChIP-seq |
| ☒ | ☐ Flow cytometry |
| ☒ | ☐ MRI-based neuroimaging |

# Eukaryotic cell lines

Policy information about cell lines and Sex and Gender in Research

| | |
| --- | --- |
| Cell line source(s) | HEK-293T cells were purchased from Millipore Sigma. |
| Authentication | Cells were authenticated by Millipore Sigma prior to purchase. |
| Mycoplasma contamination | All cells tested negative for mycoplasma contamination. |
| Commonly misidentified lines (See ICLAC register) | *Name any commonly misidentified cell lines used in the study and provide a rationale for their use.* |
